# Cluster Assembled Silicon-Lithium Nanostructures: A Nanowire Confined Inside a Carbon Nanotube

**DOI:** 10.3389/fchem.2021.767421

**Published:** 2021-11-12

**Authors:** Walter Orellana, Ricardo Pino-Rios, Osvaldo Yañez, Alejandro Vásquez-Espinal, Francesca Peccati, Julia Contreras-García, Carlos Cardenas, William Tiznado

**Affiliations:** ^1^ Departamento de Ciencias Físicas, Universidad Andres Bello, Santiago, Chile; ^2^ Laboratorio de Química Teórica, Facultad de Química y Biología, Universidad de Santiago de Chile (USACH), Santiago, Chile; ^3^ Center of New Drugs for Hypertension (CENDHY), Santiago, Chile; ^4^ Department of Pharmaceutical Science and Technology, School of Chemical and Pharmaceutical Sciences, Universidad de Chile, Santiago, Chile; ^5^ Departamento de Ciencias Químicas, Computational and Theoretical Chemistry Group, Facultad de Ciencias Exactas, Universidad Andres Bello, Santiago, Chile; ^6^ Center for Cooperative Research in Biosciences (CIC bioGUNE), Basque Research and Technology Alliance (BRTA), Bizkaia Technology Park, Derio, Spain; ^7^ Sorbonne Universités, UPMC and CNRS, Laboratoire de Chimie Théorique (LCT), 75005, Paris, France; ^8^ Departamento de Física, Facultad de Ciencias, Universidad de Chile, Santiago, Chile; ^9^ Centro para el Desarrollo de la Nanociencias y Nanotecnologia, CEDENNA, Avenida Ecuador, Santiago, Chile

**Keywords:** nanowire, density functional theory, silicon-lithium clusters, carbon nanotube, metallic character

## Abstract

We computationally explore an alternative to stabilize one-dimensional (1D) silicon-lithium nanowires (NWs). The Li_12_Si_9_ Zintl phase exhibits the NW [Li6Si5]∞1 , combined with Y-shaped Si_4_ structures. Interestingly, this NW could be assembled from the stacking of the Li_6_Si_5_ aromatic cluster. The [Li6Si5]∞1 @CNT nanocomposite has been investigated with density functional theory (DFT), including molecular dynamics simulations and electronic structure calculations. We found that van der Waals interaction between Li’s and CNT’s walls is relevant for stabilizing this hybrid nanocomposite. This work suggests that nanostructured confinement (within CNTs) may be an alternative to stabilize this free NW, cleaning its properties regarding Li_12_Si_9_ solid phase, i.e., metallic character, concerning the perturbation provided by their environment in the Li_12_Si_7_ compound.

## Introduction

The insertion of inorganic materials into single-walled carbon nanotubes (SWCNTs), hereinafter identified simply as CNT, enables the encapsulation of extreme nanowires (NWs) with diameters comparable to a unit cell of the parent material (Green, 1998; Sloan et al., 2002; Spencer et al., 2014). Although NWs of similar diameter can be produced using several templates, such as zeolites (Derouane, 1998), mesoporous phases (Alba-Simionesco et al., 2006; Ke et al., 2009), and metal-organic framework (MOF) (Lu et al., 2012) type materials, CNTs present many advantages as templates; they are atomically smooth, electron transparent, readily available, and can be filled by bulk infiltration to create milligram quantities of encapsulated nanowires, at least on a laboratory scale. Thus, encapsulated NW-CNT are scientifically interesting not only on their own but also as precursors to a wide range of other extreme nanowire materials.

In 2016, Ivanov et al. published a theoretical prediction of helix-shaped lithium-phosphorus nanowires encapsulated into single-walled carbon nanotubes (LiP@CNTs) (Ivanov et al., 2016). Note that helix-shaped Li_n_P_n_ clusters (*n* = 5–9) had previously been reported as global minimum structures (Ivanov et al., 2012). Some solid phases consist of structural motifs like atomic clusters, i.e., in Zintl phases. This connection brings consistency to the use of models based on stable clusters to generate NWs inside nanotubes, as proposed in Ivanov’s work (Ivanov et al., 2012). The study of these clusters inside CNTs can provide relevant information about these hybrid materials, for example, about their viability (stability analysis), their structural characteristics (geometry analysis), their physical and chemical properties (analysis of their electronic structure).

**Scheme 1:**
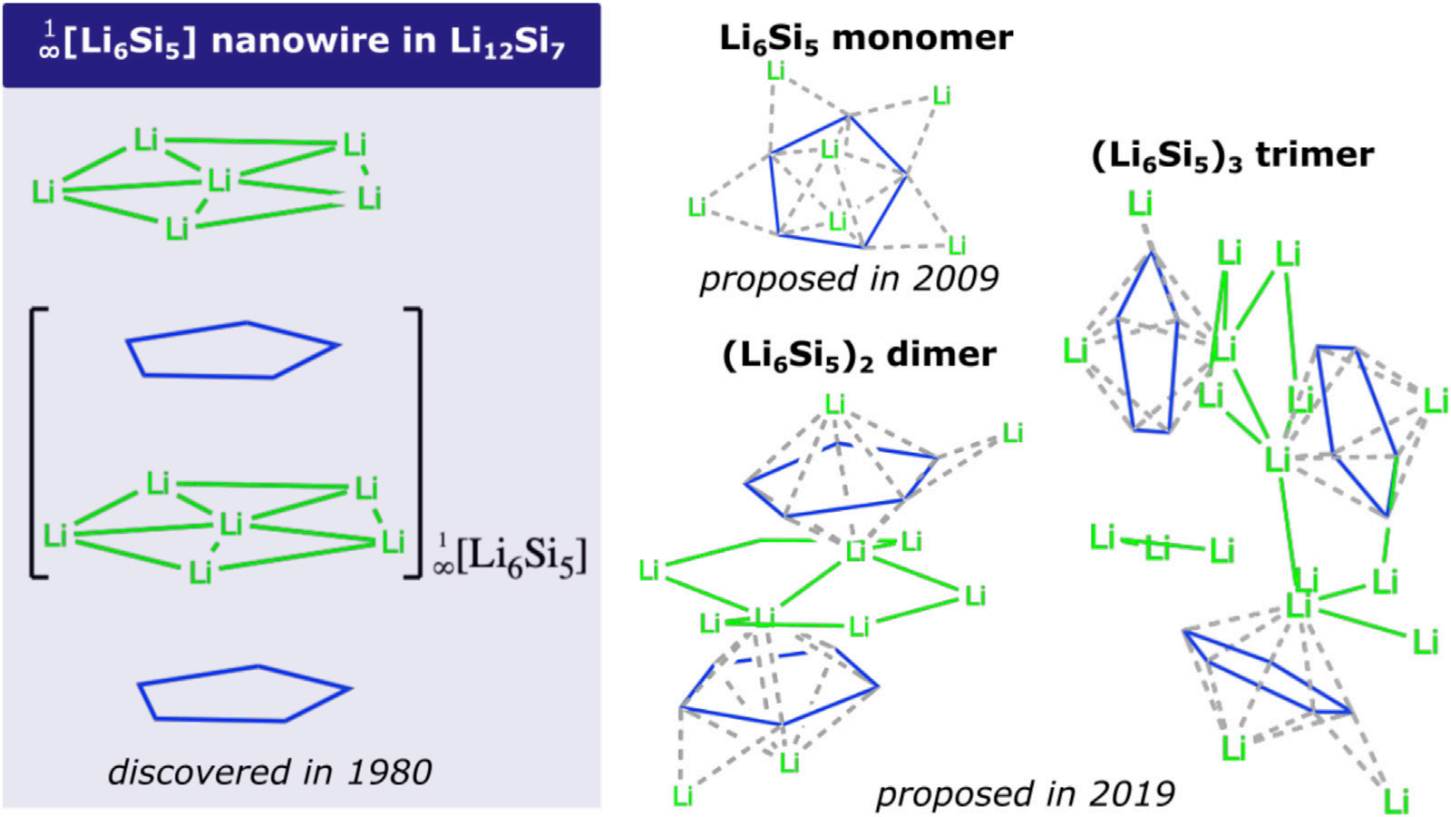
Structures of [Li6Si5]∞1 NW, Li_6_Si_5_, Li_12_Si_10_, and Li_18_Si_15_ global minima structures, where the square brackets show a repeating unit.

Due to its excellent energy storage capacity, Si has been extensively studied experimentally as a negative electrode material for Li-ion batteries (Gao et al., 2001; Ryu et al., 2004; Li et al., 2006, Li et al., 2008, Li et al., 2009; Obrovac et al., 2007; Song et al., 2014; Shin et al., 2020). Hence, Si lithifies at high temperature (415°C) in a LiCl-KCl melt, identifying potential plateaus evidencing the crystalline phases Li_12_Si_7_, Li_7_Si_3_, Li_13_Si_4_, and Li_22_Si_5_ (Wen and Huggins, 1981). In particular, the binary (non-paramagnetic) Zintl-type Li_12_Si_7_ silicide contains semi-infinite sandwich-like [Li6Si5]∞1 linear chains, consisting of Si_5_ pentagons intercalated with Li atoms (see Scheme 1). Note that the unit cell of the Zintl Li_12_Si_7_ phase has been rationalized (Nesper, 1990; Chevrier et al., 2010; Köster et al., 2011; Kuhn et al., 2011a, Kuhn et al., 2011b) as (Li_6_^6+^[Si_5_]_6_)_2_ (Li_12_^10+^[Si_4_]_10_)_2_, with two well-defined silicon moieties: planar Si_5_ rings and the Y-shaped Si_4_ moiety. Such a structural pattern is justified by assigning 26 electrons (20 from 6Si + 6 from 6Li) to the Si_5_^6-^ ring, favoring Hückel’s aromaticity (Hückel, 1930, Hückel, 1931a, Hückel, 1931b; Zhao et al., 2017). This aromatic character is supported by experimental evidence of an upfield shift (to -17.2 ppm) of Li (at the center of the Li_6_ fragment in Scheme 1) in the corresponding magic angle NMR (MAS) spectrum (Kuhn et al., 2011b; Köster et al., 2011). It is noteworthy that the Si_5_^6-^ structural motif is also present in the ternary compound Li_8_MgSi_6_ (Nesper et al., 1986a). On the other hand, at the cluster level, our group has identified that the global minimum (GM) of the Li_6_Si_5_ cluster, consists of an aromatic Si_5_^6-^ pentagon surrounded by 6 Li^+^ counterions (Tiznado et al., 2009; Perez-Peralta et al., 2011; Contreras et al., 2013; Vásquez-Espinal et al., 2018). More recently, we have identified the GM structures of the oligomers (Li_6_Si_5_)_2_ and (Li_6_Si_5_)_3_ (Yañez et al., 2019b; Manrique-de-la-Cuba et al., 2021), which also consist of aromatic Si_5_ rings surrounded by Li’s (see Scheme 1). However, the stacking of Li_6_Si_5_ units does not tend to form the nanowire identified in Li_12_Si_7_, suggesting that Li_12_^10+^[Si_4_]^10-^ component (with the Y-shaped Si_4_ moiety) contributes decisively to the stabilization of this NW. In mentioned cluster studies, explorations of the potential energy surface have been performed by hybrid methods, including genetic algorithms (Yañez et al., 2019a, Yañez et al., 2020).

To build new class of materials with desirable properties, using atomic clusters instead of atoms as building blocks, is a remarkable possibility. However, it requires that atomic clusters must retain their identity when assembled, as Khanna and Jena first outlined when they coined the word “cluster-assembled materials” (CAMs) (Khanna and Jena, 1992). These authors argued that the clusters’ coupling would have a unique effect on both the assembled material’s electronic structure and mechanical properties, which is not possible when the assembly blocks are atoms (Khanna and Jena, 1995; Jena et al., 1996; Jena and Khanna, 1996; Claridge et al., 2009; Jena and Sun, 2018). For a more detailed and timely overview of advances in the assembly of materials from clusters, please refer to the following reviews: (Chakraborty and Pradeep, 2017; Jena and Sun, 2018; Pinkard et al., 2018; Zheng et al., 2019; Doud et al., 2020).

Given the above background, here we evaluated, *in silico*, the stability of the isolated [Li6Si5]∞1 NW, as well as its electronic properties. In addition, we studied the hybrid material consisting of the NW confined in a CNT. The latter focused on identifying alternative ways to stabilize this conformation and to evaluate the effect of this association on NW electronic properties. Our density functional theory (DFT) calculations demonstrate that Li-Si@CNTs hybrid systems have excellent stability and thus have potential for experimental realization.

## Computational Methods

In the finite models (clusters), geometry optimizations and frequency calculations were performed at the PBE0 (Adamo and Barone, 1999)/Def2TZVP(Weigend and Ahlrichs, 2005) level with the Gaussian 16 program (M. J. Frisch, G. W. Trucks, H. B. Schlegel et al., 2016).

For the solid-state study, we performed first-principles calculations based on DFT (Sham and Kohn, 1966; Kohn et al., 1996) as implemented in the Vienna Ab Initio Simulation Package (VASP) (Kresse and Furthmüller, 1996). The exchange-correlation energies were calculated at PBE-D3 level (Ernzerhof and Perdew, 1998; Grimme et al., 2010). Plane-wave basis set with a kinetic energy cutoff of 400 eV, and the projector augmented-wave method for the core-valence interaction was employed (Blöchl et al., 1994). The [Li6Si5]∞1 NWs were simulated in a large unit cell with volume (20 × 20 × *z*_0_) Å^3^, considering periodic boundary conditions along the NW direction, where *z*_0_ is the periodicity. Within this supercell, the lateral distance between NW images is set to 15 Å. We use a (1 × 1 × 10) Monkhorst-Pack k-point mesh (Monkhorst and Pack, 1976). We also study finite (Li_6_Si_5_)_4_ structures in the free space, also inside both an armchair and a zigzag single-walled carbon nanotube (SWCNTs). We consider armchair CNTs with chiral indexes (8,8), (9,9), and (10,10), which have diameters of 10.93, 12.27, and 13.63 Å, and zigzag CNTs with chiral indexes (14,0), (15,0), and (16,0) which have diameters of 11.04, 11.80, and 12.59 Å, respectively. For (Li_6_Si_5_)_4_@CNT simulation, (22 × 22 × *z*_0_) Å^3^ volume was used, where *z*_0_ is the periodicity chosen for the CNTs. All studied structures were allowed to freely relax without any constraint until forces on each atom were smaller than 25 meV/Å. To gain insights on the stability of the NW models in the free space and inside the SWCNTs, we performed Born-Oppenheimer ab initio molecular dynamics (BO-AIMD) simulations within the NVT ensemble at different temperatures, over a total simulation time of 10 ps, considering a time step of 1 fs.

## Results and Discussion

### Finite Model Tests to Estimate the Optimal Width of SWNTS

The first question that arises is which is the optimal SWCNT width to favor the [Li6Si5]∞1 NW grown? This is a relevant question, considering that the electronic structure of group 1 elements, such as Li, is particularly sensitive to confinement (Robles-Navarro et al., 2020). To get an idea of the nanotube widths to be considered in our study, we first performed a finite model analysis. This model consists of [n]cyclacenes (*n* = 13–20) in their optimal structure (at the PBE0/Def2TZVP level), covering the diameter range from 10.2 to 15.6 Å. Then the Li_7_Si_5_^+^ cluster was placed, centering it on emulating the growth pattern towards the nanowire (see Scheme 1). We choose the star-shaped D_5h_-Li_7_Si_5_^+^ cluster as a suitable model for projecting the nanowire inside the CNT due to its high symmetry and its analogy in electronic structure with the Li_6_Si_5_ unit. In this study, we have kept the [n] cyclacene structure rigid, allowing only the optimization of the Li_7_Si_5_^+^ structure (at the PBE0/Def2TZVP level).

**FIGURE 1:**
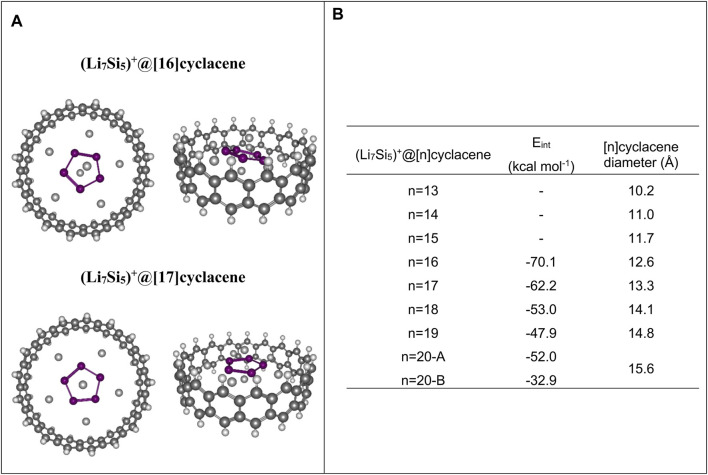
**(A)** Top-and side-views of optimized structures of Li_7_Si_5_^+^ inside both [16]cyclacene and [17]cyclacene at PBE0/def2-TZVP level. **(B)** Li_7_Si_5_^+^ and [n]cyclacene interaction energy (E_int_), number of hexagonal rings and diameter (in Å) of the [n]cyclacene.

In the case of small [n]cyclacenes (*n* = 13–15), Li_7_Si_5_^+^ cluster undergoes noticeable changes in the optimization process due to the confinement effects. In contrast, when [n]cyclocenes with *n* = 16–20 are used, the Li_7_Si_5_^+^ cluster maintains its structure at the end of the optimization process, leading to the best interaction energy, [E_int_ = E (Li_7_Si_5_^+^at[n]cyclacene)-(E (Li_7_Si_5_)^+^E ([n]cyclacene))], with [16]cyclacene (-70.1 kcal mol^−1^ at PBE0/Def2TZVP level). Since this analysis is only a reference for estimating the most suitable nanotube diameters to explore in the periodic calculations, we have not included basis set superposition error (BSSE) corrections. The most stable structures, as well as the E_int_, are shown in Figure 1. The structures for the other complexes are shown in Supplementary Figure S1 and their Cartesian coordinates in Supplementary Table S1. These results guided us to use CNTs with diameters in the range of 11–14 Å in next steps of our research.

### Insights on the stability of free [Li6Si5]∞1 NW.

**FIGURE 2:**
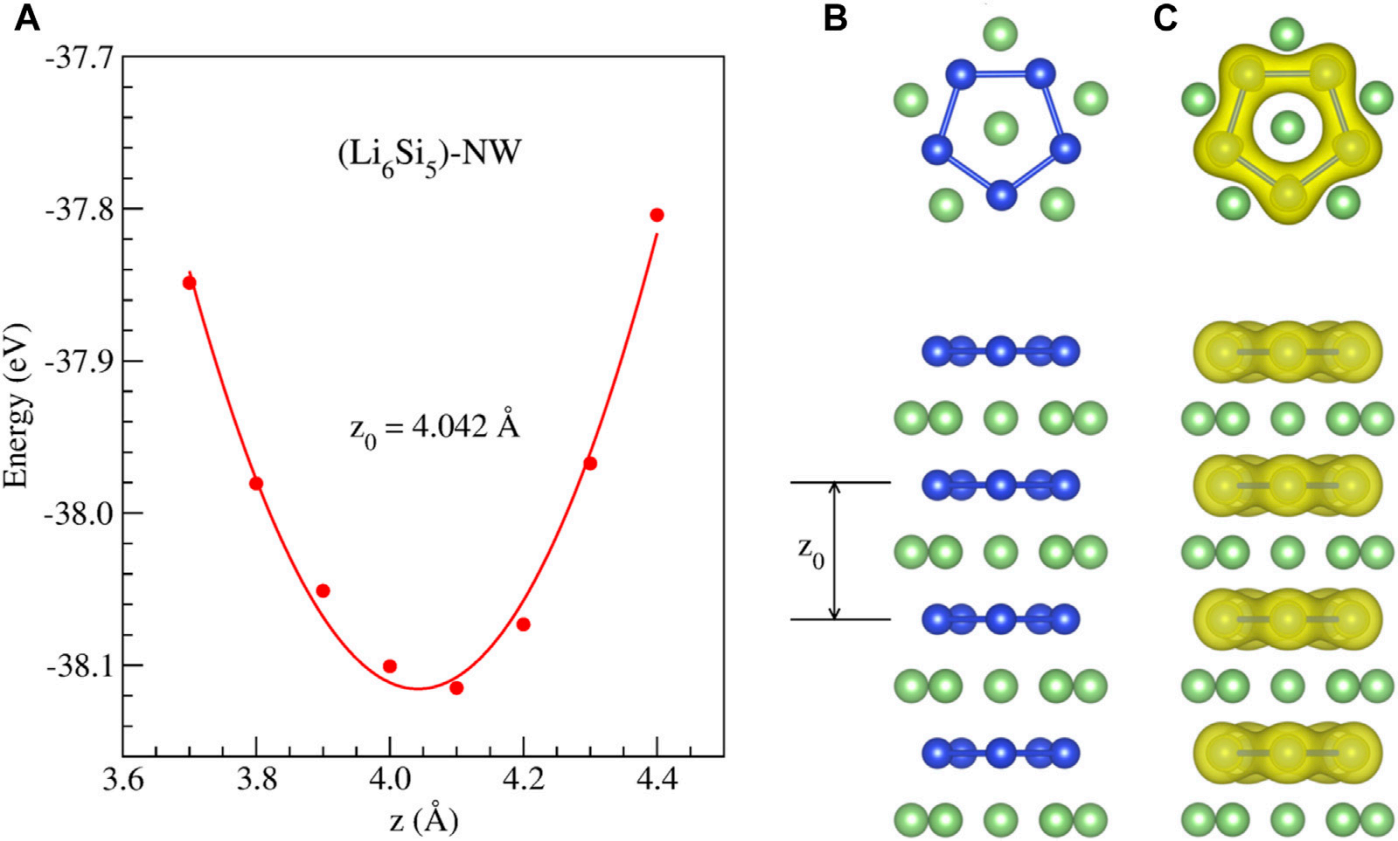
Stability of the [Li6Si5]∞1 isolated model (Li_6_Si_5_-NW) obtained from periodic DFT calculations: **(A)** Energy as a function of the distance between Li_6_Si_5_ units, **(B)** top and side views of the equilibrium geometry, **(C)** electronic density distribution for the isosurface at 0.05 *e*/Å^3^.

We first studied the stability of periodically repeated Li_6_Si_5_ units (Li_6_Si_5_-NW), which are stacked along the *z* direction, forming a one-dimensional structure, as shown in Figure 2.

**FIGURE 3:**
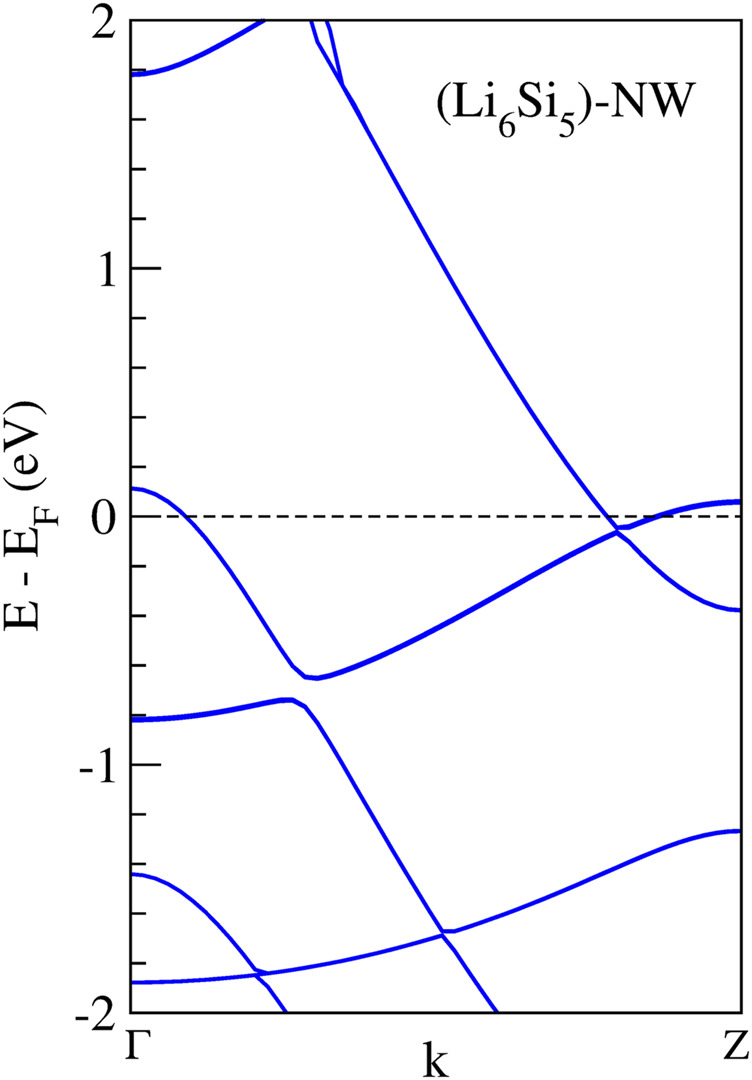
Band structures calculation of the Li_6_Si_5_-NW in the unit cell. The dashed line indicates the Fermi energy.

We found a stable structure with an equilibrium distance between Si_5_ rings of 4.04 Å which is the periodicity of the Li_6_Si_5_ unit cell. In the equilibrium geometry, the distance between Li atoms of the border is 3.33 Å while the distance with respect to the center one is 2.83 Å. The Si-Si distance between neighboring atoms is 2.37 Å, very close to the ones in Li_6_Si_5_ monomer (between 2.30 and 2.35 Å). Our computations of the electronic band structure of the Li_6_Si_5_-NW in the primitive unit cell suggest a metallic character (see Figure 3). Note that the bandgaps of Li_12_Si_7_ was reported from conductivity-temperature experimental measurements and found to be 0.6 eV (Nesper et al., 1986b).

**FIGURE 4:**
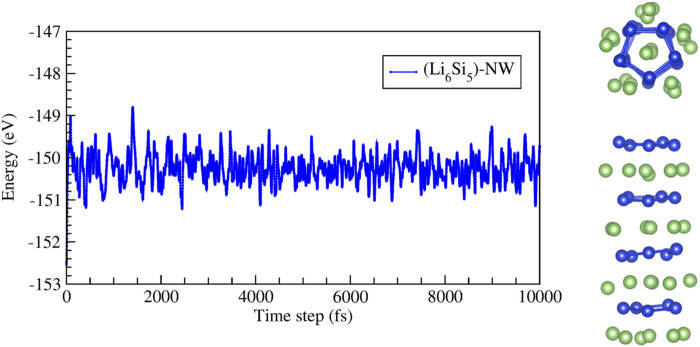
Energy as a function of time for the molecular dynamic simulations at 500 K of the infinite Si_5_Li_6_ nanowire. The unit cell for this simulation considers four Si_5_Li_6_ units. The right images show top and side views of a snapshot taken at 10,000 fs

The stability of the Li_6_Si_5_-NW was also verified by BO-AIMD simulations at 300 K and 500K, during a simulation time of 10 ps. The simulation was performed by considering four Li_6_Si_5_ units in the periodic unit cell, as shown in Figure 4. We observe that at 500 K the Si_5_Li_6_ NW preserves its stability, showing energy fluctuations of around 2 eV. Supporting information contains short movies extracted from the BO-AIMD simulations.

### Stability of the Li_6_Si_5_-NW Inside the CNTs

**FIGURE 5:**
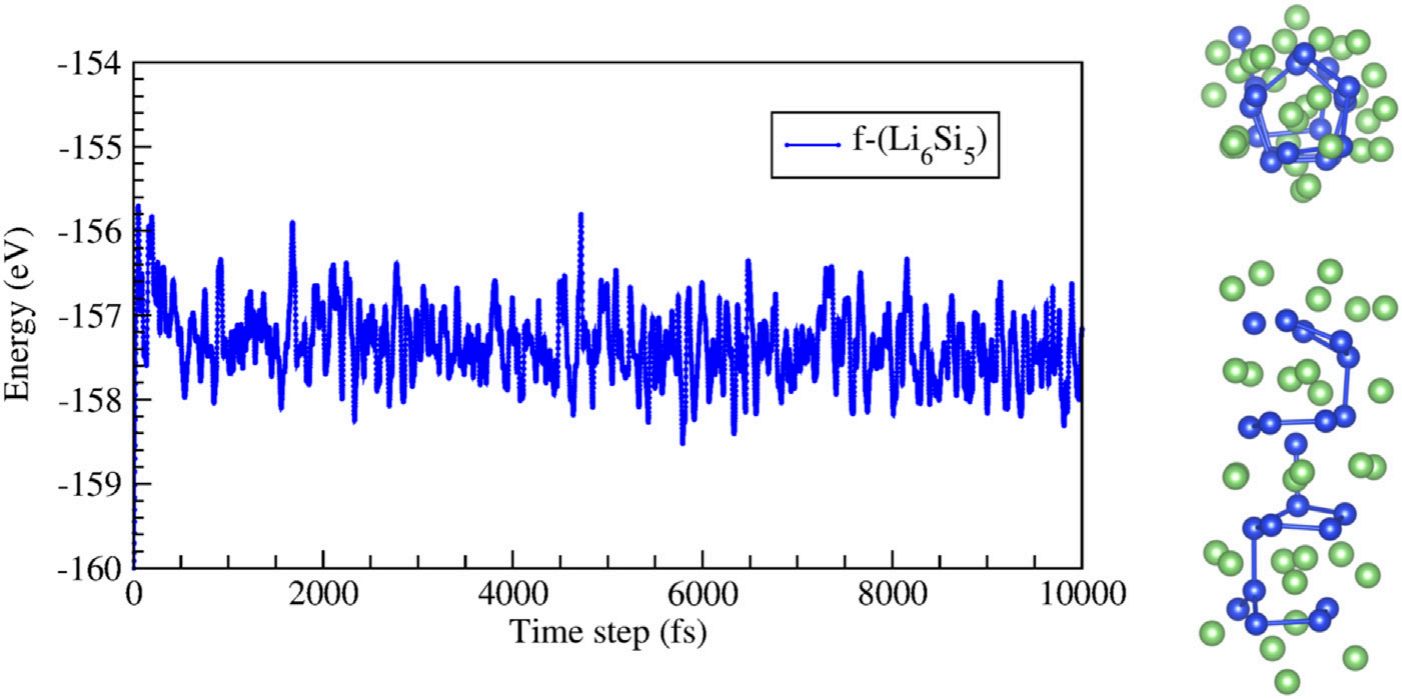
Energy as a function of time for molecular dynamic simulations at 500 K for the finite Li_6_Si_5_ structure (f-Li_6_Si_5_). The right images show top and side views of a snapshot taken at 10,000 fs

Next, we studied a finite Li_6_Si_5_ structure (f-Li_6_Si_5_) in the free space and encapsulated it inside both armchair and zigzag carbon nanotubes (f-Li_6_Si_5_@CNT). For the f-Li_6_Si_5_ structure, we consider four Si_5_ rings surrounded by five Li_6_ moieties. BO-AIMD simulations provide insights on the stability of the f-Li_6_Si_5_ system in the free space at 300 K and 500 K. We find that at 300 K, the f-Li_6_Si_5_ structure preserves its stability. Still, at 500 K, it tends to form Si-Si bonds between adjacent Si rings without losing its one-dimensional array, as shown in Figure 5. However, it is important to note that this model does not have the exact stoichiometry of NW because, to maintain symmetry, an extra Li_6_ unit group is added, i.e., [(Li_6_)_5_(Si_5_)_4_].

**FIGURE 6:**
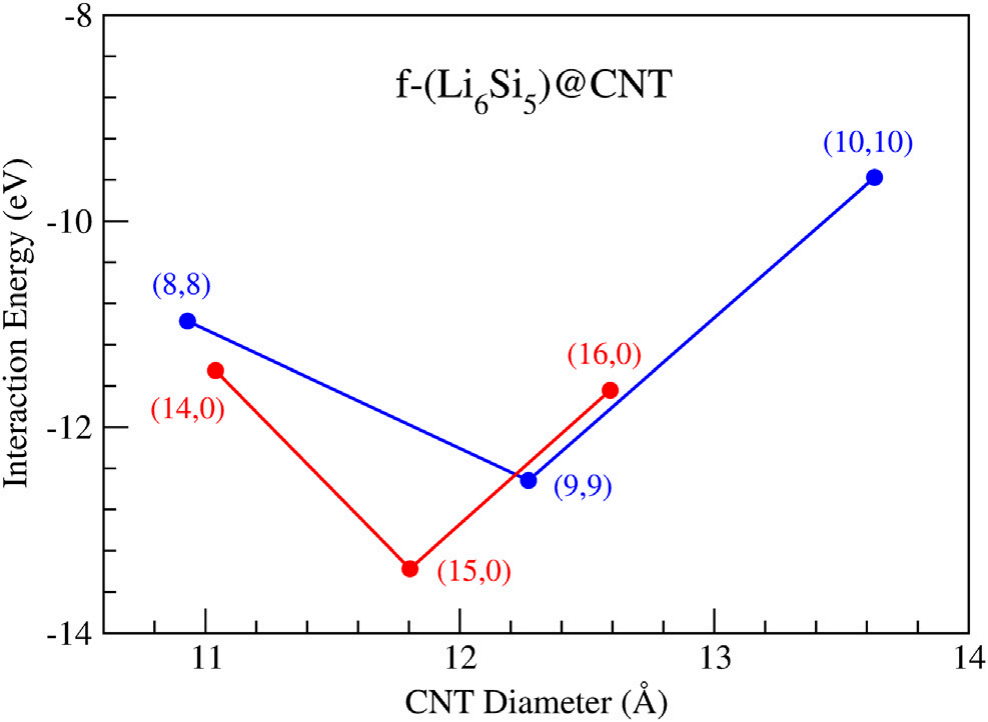
Interaction energy as a function of the nanotube diameters for the finite Li_6_Si_5_ structures inside armchair CNTs with chiral indexes (8,8), (9,9), and (10,10) and zigzag CNTs with chiral indexes (14,0), (15,0), and (16,0).

**FIGURE 7:**
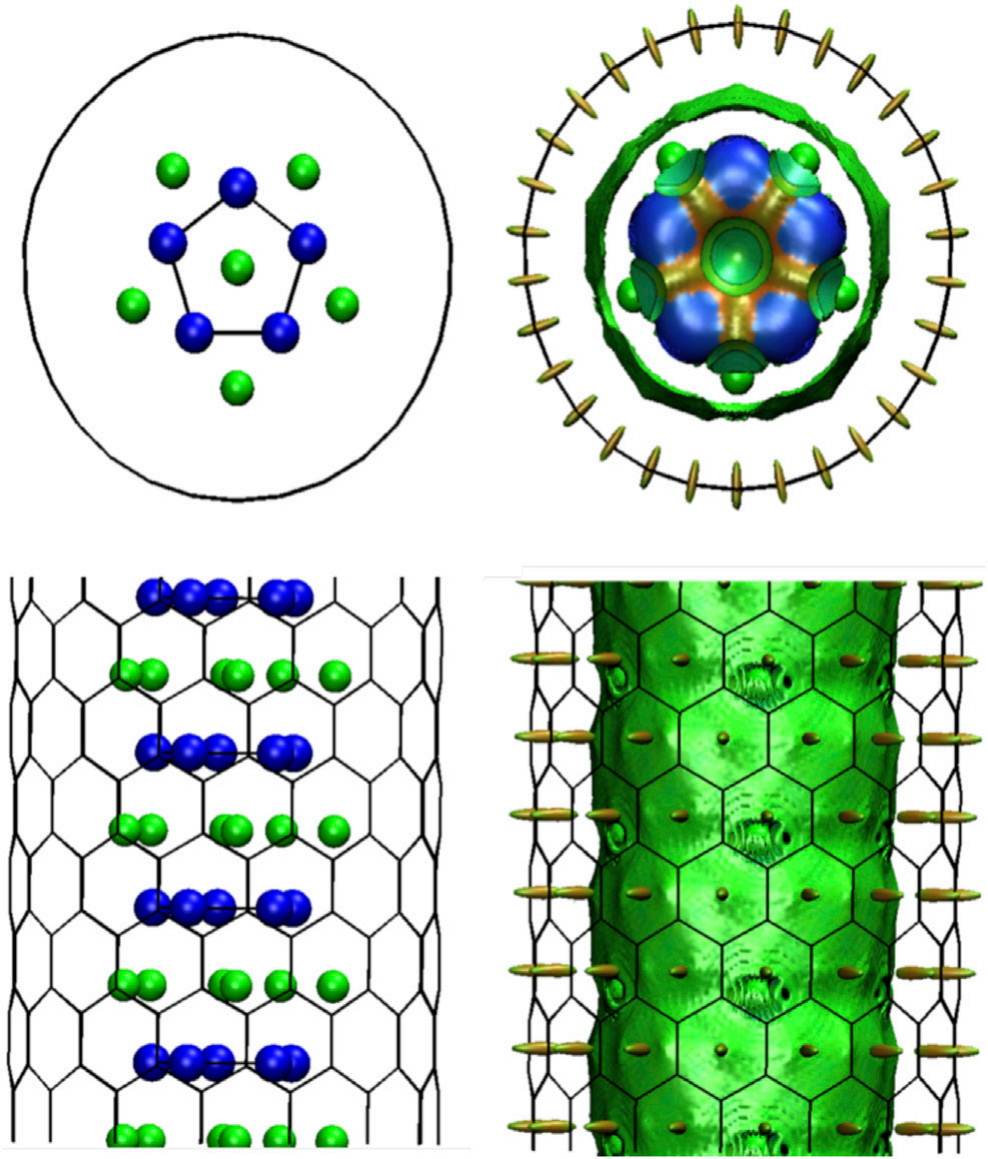
f-Li_6_Si_5_@NTC optimized structure, for zigzag CNTs with chiral indexes (15,0), with NCI surfaces (*s* = 0.3, color range: −0.03 to 0.03 a.u.). Geometries and electron density were taken from solid state computations.

To study the f-Li_6_Si_5_ structure inside the CNTs, we consider three armchair CNTs with chiral indexes of (8,8), (9,9), and (10,10), and three zigzag CNTs with chiral indexes of (14,0), (15,0), and (16,0). With this choice, we seek to find the CNT size that best accommodates the Li_6_Si_5_-NW inside. Note that we selected these CNTs according to our preliminary findings from the Li_7_Si_5_^+^@[n]cyclacene model, suggesting diameters between 12 and 15 Å. The CNTs were simulated with periodic boundary conditions along its axis with a periodicity of *z*_0_ = 30 Å. The latter allows a vacuum region for the encapsulated f-Li_6_Si_5_ structure of 14 Å, allowing the atomic movement inside the CNT. Next, we calculate the $E_{int}$ between encapsulated f-Li_6_Si_5_ and the CNTs by the equation:

$$E_{\mathrm{int}}=E_{tot}\left(f–{\mathrm{Li}}_{6}{\mathrm{Si}}_{5}@\mathrm{CNT}\right)-E_{\mathrm{tot}}\left(f–{\mathrm{Li}}_{6}{\mathrm{Si}}_{5}\right)-E_{\text{tot}}\left(\mathrm{CNT}\right),$$

where $E_{tot}\left(f–{\text{Li}}_{6}{\text{Si}}_{5}\right)$ and $E_{\text{tot}}\left(\text{CNT}\right)$ are the total energies of the isolated f-Li_6_Si_5_ and the CNT, respectively, and $E_{\text{tot}}\left(f–{\text{Li}}_{6}{\text{Si}}_{5}@\text{CNT}\right)$ is the total energy of f-Li_6_Si_5_ unit inside the CNT. Our results for the $E_{\text{int}}$ are shown in Figure 6. The armchair (9,9) and the zigzag (15,0) CNTs of 12.3 and 11.8 Å in diameter, respectively, exhibit the more stabilizing $E_{\text{int}}$ , being the best candidates to accommodate the f-Li_6_Si_5_ inside. In addition, the f-Li_6_Si_5_ is better stabilized inside the zigzag (15,0) CNT than inside the armchair (9,9) CNT by 0.9 eV. Noteworthy, the larger-diameter CNTs are energetically less favorable to encapsulate the f-Li_6_Si_5_, as shown in Figure 6, in agreement with the Li_7_Si_5_^+^@[n]cyclacene model. This behavior is presumable due to the van der Waals (vdW) interaction between the f-Li_6_Si_5_ and the CNT internal walls, stabilizing the system. The non-covalent interaction index (NCI) plots confirm the non-covalent character of f-Li_6_Si_5_ with the CNT [f-Li_6_Si_5_ inside the zigzag (15,0) CNT]. In the NCI method (Johnson et al., 2010; Contreras-García et al., 2011), an isosurface of the reduced density gradient (s) is colored with the density times the sign of the second eigenvalue of the electron density Hessian matrix, λ_2_, to distinguish between attractive and repulsive interactions. The following color code is used: blue for attractive such as hydrogen bonds, green for very weak interactions such as vdW and red for steric repulsion. Figure 7 depicts the second one (vdW) between f-Li_6_Si_5_ and the walls of the CNT.

**FIGURE 8:**
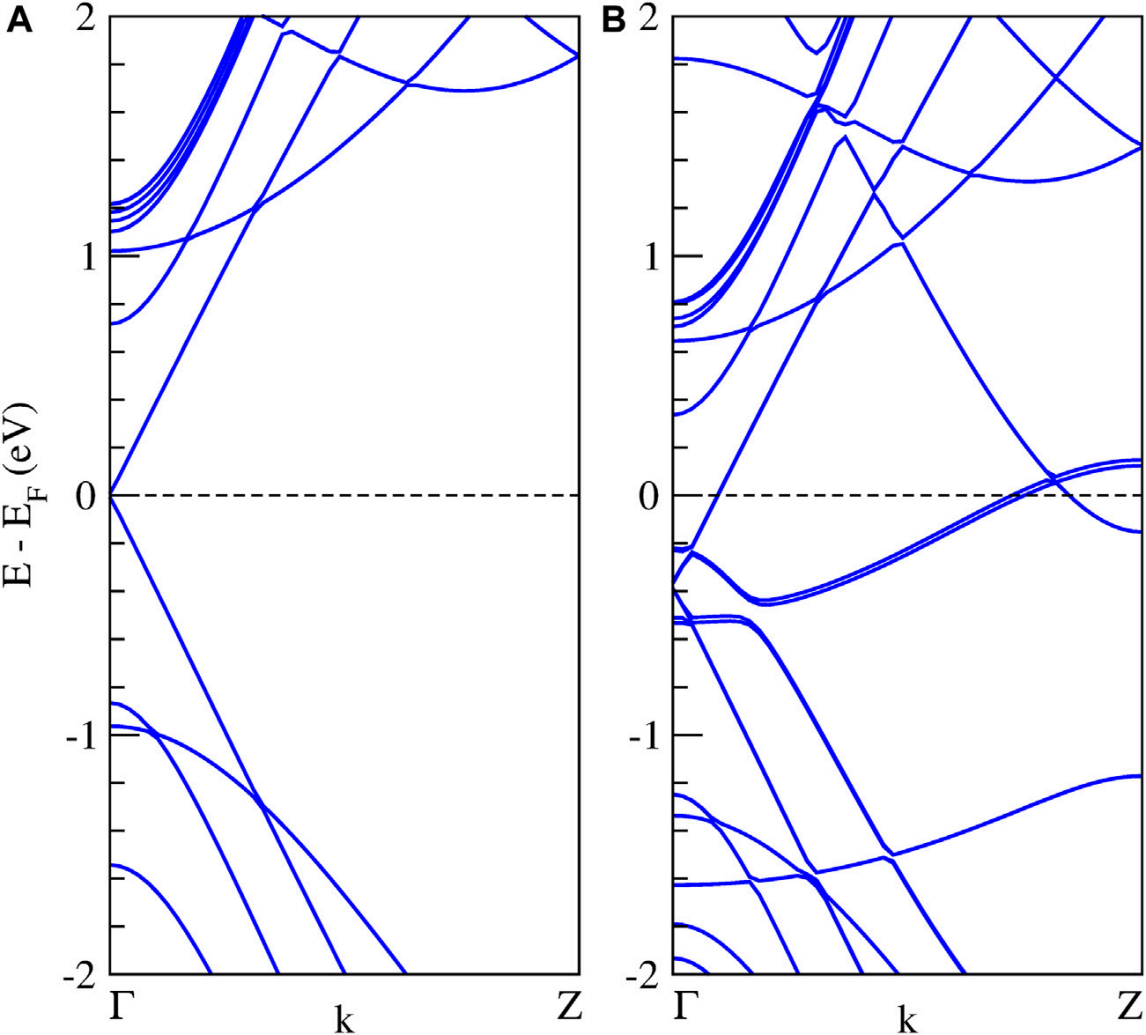
Band structure calculations of the infinite Li_6_Si_5_-NW inside the zigzag carbon nanotubes, **(A)** the isolated (15,0) CNT, and **(B)** the Li_6_Si_5_-NW@CNT system. The dashed line indicates the Fermi energy.

We also calculate the band structure of the Li_6_Si_5_-NW inside the zigzag (15,0) CNT. It is important to note that the unit cells of the Li_6_Si_5_-NW and the CNT have a mismatch of 5.7%, which means that the Li_6_Si_5_-NW is not in its equilibrium geometry in the Li_6_Si_5_-NW@CNT unit cell, where the distance between the Si_5_ rings increases by 0.23 Å. However, this mismatch is relatively small and should not affect the electronic properties of the system. For the isolated CNT we find a small bandgap of 0.02 eV as shown in Figure 8A, which is in good agreement with the measured value of 0.029 ± 0.004 eV (Ouyang et al., 2001). Whereas the Li_6_Si_5_-NW@CNT system exhibits metallic properties as shown Figure 8B, suggesting that the Li_6_Si_5_-NW would preserve its electronic properties inside the CNT as can be compared with Figure 3.

**FIGURE 9:**
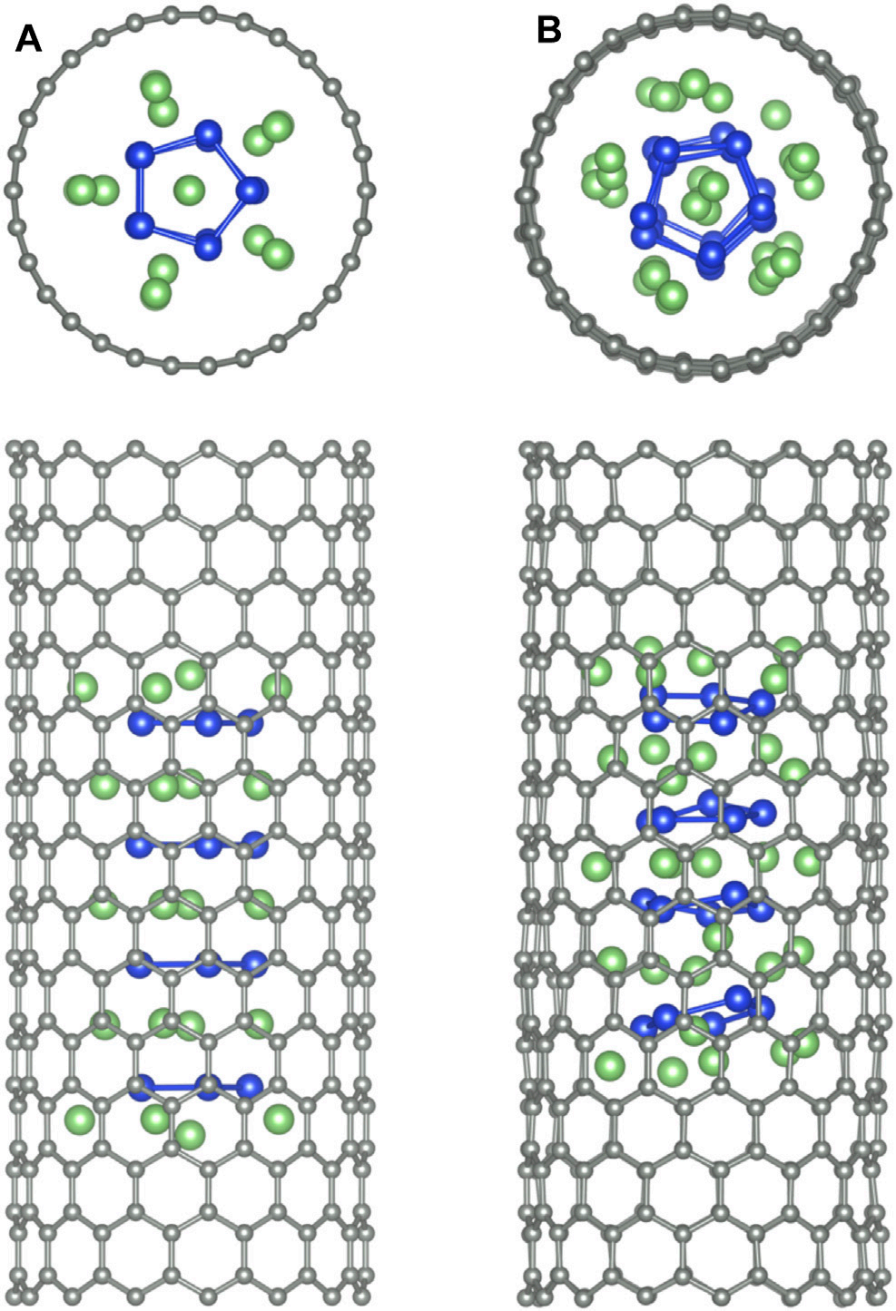
The f-Li_6_Si_5_ structure inside a zigzag (15,0) CNT. **(A)** Top and side views of the equilibrium geometry at 0 K. **(B)** Top and side views of a snapshot taken at 2000 fs of BO-AIMD simulation at 300 K. The periodicity of the CNT along its axis is z_0_ = 30 Å.

Finally, we study the stability of the f-Li_6_Si_5_ structure inside both zigzag (15,0) and armchair (9,9) CNTs by performing BO-AIMD simulations. Figure 9A shows the equilibrium geometry of the f-Si_5_Li_6_ structure inside the (15,0) CNT. We find that the structure remains almost unchanged with respect to f-Si_5_Li_6_ in the free space, showing that the CNT would have a small influence in the Li_6_Si_5_ NW stability. We only note a small displacement of the Li ions at the extreme of the f-Li_6_Si_5_ structure which move toward the CNT wall. The integrity of the f-Li_6_Si_5_ structure inside the (15,0) and (9,9) CNTs was investigated by BO-AIMD simulations at 300 K. We find that the f-Si_5_Li_6_ structure preserves its stability where the Li ions move around the Si_5_ ring without detaching. Similar results are found for the f-Li_6_Si_5_ structure inside the armchair (9,9) CNT, indicating that the formation and stability of the Li_6_Si_5_ NW inside the CNTs is independent of its chirality. This result suggests that Li_6_ Si_5_-NW are likely to form inside CNTs in a compact form, which would allow efficient storage of Li ions into CNTs mediated by Si_5_ rings. Supplementary Figure S2 shows the variation of the total energy for the BO-AIMD simulation of f-Li_6_Si_5_ inside the (15,0) and (9,9) CNTs at 300 K. We observe energy fluctuation of around 5 eV in both CNTs, preserving the stability of the f-Li_6_Si_5_ structure.

## Conclusion

Using periodic DFT calculations and Born-Oppenheimer *ab initio* molecular dynamic simulations, we have shown that Li_6_Si_5_ units can be stacked one above the other, forming a one-dimensional structure linked together by Coulomb interactions. This study complements previous findings, where we demonstrated that Li_6_Si_5_, (Li_6_Si_5_)_2_, and (Li_6_Si_5_)_3_ lowest energy structures contain one, two, and three Si_5_^6-^ aromatic rings stabilized by Li^+^ counterions. Additionally, the [Li6Si5]∞1 nanowire was identified in the Zintl Li_12_Si_7_ compound but coexisting with Y-shaped Si_4_ moieties. In this case, we support the stability of the isolated Si-Li-nanowire—additionally, the relaxed structure (at room temperature) exhibits metallic characteristics.

We also found that finite (Li_6_Si_5_)_4_ systems are stable inside both armchair and zigzag carbon nanotubes of around 12 Å in diameter, preserving its stability at room temperature, supporting the viable formation of Li_6_Si_5_-NW inside the CNTs. Interestingly, the Li_6_Si_5_-NW@CNTs hybrid nanocomposite maintains the metallic character. Finally, in the Li_6_Si_5_-NW, the Li_6_Si_5_ units are connected by strong electrostatic interactions (Si_5_^6-^ aromatic pentagons intercalated with the Li_6_^6+^ moiety) in agreement with the Zintl ion concept. In the [Li_6_Si_5_-NW]@CNTs, NCI predicts that Li_6_Si_5_-NW interacts with the CNT walls by van der Waals interactions Frisch et al., 2016.

## Data Availability

The original contributions presented in the study are included in the article/Supplementary Material, further inquiries can be directed to the corresponding author.
