# Supplementary material for: Cluster Assembled Silicon-Lithium Nanostructures: A Nanowire Confined Inside a Carbon Nanotube
Source: Front Chem. 2021 Nov 12;9:767421. doi: 10.3389/fchem.2021.767421 (PMC8633442; doi:10.3389/fchem.2021.767421)
Supplement: Supplementary file 2 [file DataSheet1.PDF]

# *Supplementary Material*

**Cluster Assembled Silicon-Lithium Nanostructures: A Nanowire Confined Inside a Carbon Nanotube**

**Walter Orellana[1], Ricardo Pino-Rios[2], Osvaldo Yañez[3,4], Alejandro Vásquez-Espinal[5], Francesca Peccati[6], Julia Contreras-García[7], Carlos Cardenas[8,9] and William Tiznado[5*]**

[1]*Departamento de Ciencias Físicas, Universidad Andres Bello, Sazié 2212, Santiago 0370136, Chile.*
[2]*Laboratorio de Química Teórica, Facultad de Química y Biología, Universidad de Santiago de Chile (USACH), 8320000 Santiago, Chile.*
[3]*Center of New Drugs for Hypertension (CENDHY), 8380494 Santiago, Chile.*
[4]*Department of Pharmaceutical Science and Technology, School of Chemical and Pharmaceutical Sciences, Universidad de Chile, 8380494 Santiago, Chile.*
[5]*Computational and Theoretical Chemistry Group, Departamento de Ciencias Químicas, Facultad de Ciencias Exactas, Universidad Andres Bello, República 498, 8370035 Santiago, Chile*
[6]*Center for Cooperative Research in Biosciences (CIC bioGUNE), Basque Research and Technology Alliance (BRTA), Bizkaia Technology Park, 48160 Derio, Spain*
[7]*Sorbonne Universités, UPMC and CNRS, Laboratoire de Chimie Théorique (LCT), 75005 Paris, France.*
[8]*Departamento de Física, Facultad de Ciencias, Universidad de Chile, Casilla 653 Santiago, Chile.*
[9]*Centro para el Desarrollo de la Nanociencias y Nanotecnologia, CEDENNA, Avenida Ecuador 3493, 9170124 Santiago, Chile.*

***Correspondence:**
*Corresponding Author*

*wtiznado@unab.cl*

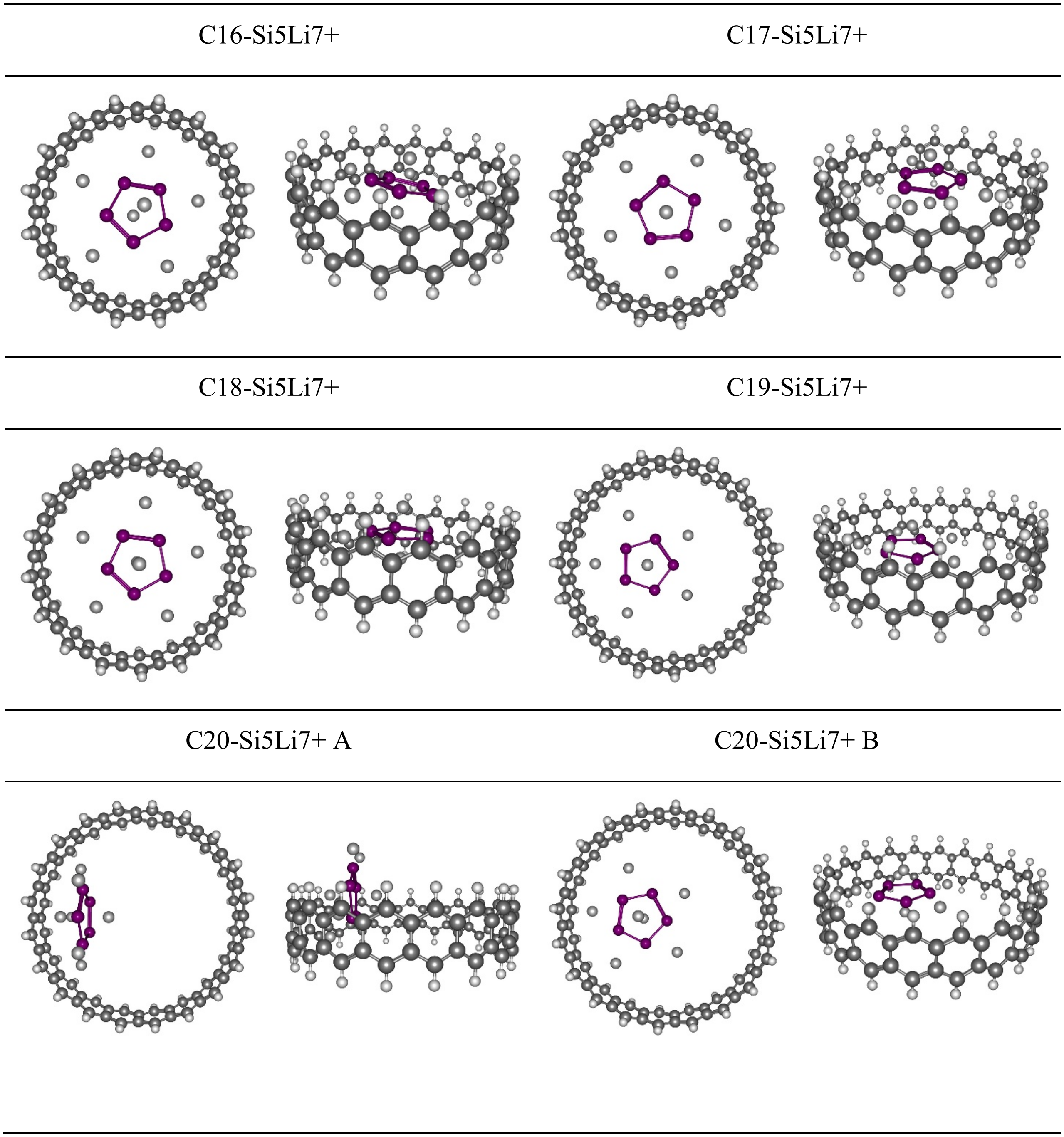


**Figure S1.** (a) Top-and side-views of optimized structures of $Li_7Si_5^+$ inside both [n]cyclacene (n=16-20) at PBE0/def2-TZVP level.

**Table S1.** Cartesian coordinates of the structures reported in Fig. S1.All the optimizations were performed in singlet states.

| $Si_5Li_7^+$@[16]cyclacene | | | | $Si_5Li_7^+$@[17]cyclacene | | | |
|---|---|---|---|---|---|---|---|
| 6 | 6.133305000 | 1.323069000 | -1.398580000 | 6 | 6.730285000 | 2.431656000 | -1.664635000 |
| 6 | 5.821098000 | -2.304646000 | -0.725093000 | 6 | 7.102777000 | -1.213369000 | -0.983191000 |
| 6 | 4.490032000 | -4.358469000 | 0.734311000 | 6 | 6.267310000 | -3.496224000 | 0.504701000 |
| 6 | 3.554715000 | -5.157872000 | 1.409735000 | 6 | 5.544865000 | -4.497202000 | 1.202424000 |
| 6 | 1.310081000 | -6.123527000 | -1.412069000 | 6 | 1.200903000 | -6.669327000 | -1.497084000 |
| 6 | 0.087094000 | -6.255222000 | -0.736355000 | 6 | -0.028685000 | -6.666437000 | -0.790826000 |
| 6 | -2.319730000 | -5.814631000 | 0.724181000 | 6 | -2.352133000 | -6.005902000 | 0.722021000 |
| 6 | -3.416339000 | -5.258153000 | 1.400748000 | 6 | -3.403464000 | -5.361117000 | 1.421706000 |
| 6 | -5.168621000 | -3.549630000 | -1.417751000 | 6 | -5.114501000 | -3.591833000 | -1.355707000 |
| 6 | -5.757996000 | -2.470961000 | -0.740079000 | 6 | -5.653613000 | -2.473849000 | -0.670101000 |
| 6 | -5.828907000 | 2.323542000 | 0.727523000 | 6 | -5.648387000 | 2.345582000 | 0.771089000 |
| 6 | -5.272354000 | 3.418417000 | 1.406701000 | 6 | -5.090561000 | 3.470476000 | 1.429918000 |
| 6 | -0.092934000 | 6.275732000 | -0.724438000 | 6 | -0.111180000 | 6.601192000 | -0.838643000 |
| 6 | 2.310034000 | 5.832219000 | 0.741376000 | 6 | 2.362914000 | 6.395845000 | 0.561434000 |
| 6 | 3.404923000 | 5.274528000 | 1.419569000 | 6 | 3.546644000 | 5.963036000 | 1.211060000 |
| 6 | 5.164337000 | 3.571013000 | -1.397397000 | 6 | 5.420846000 | 4.505179000 | -1.641002000 |
| 6 | 5.751849000 | 2.491274000 | -0.720522000 | 6 | 6.188328000 | 3.513511000 | -0.979096000 |
| 6 | 5.819171000 | -2.306067000 | 0.738032000 | 6 | 7.137968000 | -1.207353000 | 0.475106000 |
| 6 | 5.260931000 | -3.402417000 | 1.413663000 | 6 | 6.828279000 | -2.407629000 | 1.163678000 |
| 6 | 3.558355000 | -5.155072000 | -1.408247000 | 6 | 5.497704000 | -4.484796000 | -1.608876000 |
| 6 | 2.478199000 | -5.743993000 | -0.732762000 | 6 | 4.592842000 | -5.321219000 | -0.907379000 |
| 6 | 0.085142000 | -6.256733000 | 0.726795000 | 6 | 0.007013000 | -6.661167000 | 0.667475000 |
| 6 | -1.141252000 | -6.161919000 | 1.402707000 | 6 | -1.204135000 | -6.445194000 | 1.372507000 |
| 6 | -3.412807000 | -5.255465000 | -1.417220000 | 6 | -3.446608000 | -5.389536000 | -1.389520000 |
| 6 | -4.370556000 | -4.484463000 | -0.740276000 | 6 | -4.351215000 | -4.547741000 | -0.694098000 |
| 6 | -5.759705000 | -2.472659000 | 0.722750000 | 6 | -5.618082000 | -2.468920000 | 0.788188000 |
| 6 | -6.176555000 | 1.143531000 | 1.403381000 | 6 | -5.967449000 | 1.180266000 | 1.459792000 |
| 6 | -5.268849000 | 3.421164000 | -1.411299000 | 6 | -5.174994000 | 3.433707000 | -1.380290000 |
| 6 | -4.497945000 | 4.377212000 | -0.731725000 | 6 | -4.407159000 | 4.430452000 | -0.725910000 |

| | | | | | | | |
|---|---|---|---|---|---|---|---|
| 6 | -0.094831000 | 6.274402000 | 0.738685000 | 6 | -0.076374000 | 6.606892000 | 0.619674000 |
| 6 | 1.129792000 | 6.178187000 | 1.417599000 | 6 | 1.185774000 | 6.624956000 | 1.265632000 |
| 6 | 3.408479000 | 5.276891000 | -1.398449000 | 6 | 3.450981000 | 5.965587000 | -1.599018000 |
| 6 | 4.364002000 | 4.505610000 | -0.720633000 | 6 | 4.526763000 | 5.312365000 | -0.945911000 |
| 6 | 5.750022000 | 2.489911000 | 0.742604000 | 6 | 6.223220000 | 3.519514000 | 0.479203000 |
| 6 | 6.129665000 | 1.320329000 | 1.419397000 | 6 | 6.809207000 | 2.413708000 | 1.145921000 |
| 6 | 5.264653000 | -3.399682000 | -1.404307000 | 6 | 6.771529000 | -2.389425000 | -1.647378000 |
| 6 | 4.491960000 | -4.357036000 | -0.728846000 | 6 | 6.231892000 | -3.502142000 | -0.953622000 |
| 6 | 2.476329000 | -5.745505000 | 0.730339000 | 6 | 4.628402000 | -5.315498000 | 0.550946000 |
| 6 | 1.306540000 | -6.126613000 | 1.405879000 | 6 | 1.238069000 | -6.661889000 | 1.314412000 |
| 6 | -1.137657000 | -6.159031000 | -1.415212000 | 6 | -1.242286000 | -6.463499000 | -1.438870000 |
| 6 | -2.317885000 | -5.813195000 | -0.738968000 | 6 | -2.387751000 | -6.010887000 | -0.736254000 |
| 6 | -4.372388000 | -4.485862000 | 0.722881000 | 6 | -4.315704000 | -4.542869000 | 0.764193000 |
| 6 | -5.172105000 | -3.552230000 | 1.400205000 | 6 | -5.062899000 | -3.555614000 | 1.455250000 |
| 6 | -6.172979000 | 1.146205000 | -1.414575000 | 6 | -6.041374000 | 1.139570000 | -1.350635000 |
| 6 | -5.827046000 | 2.324939000 | -0.735589000 | 6 | -5.683760000 | 2.340607000 | -0.687205000 |
| 6 | -4.499552000 | 4.376045000 | 0.731031000 | 6 | -4.372291000 | 4.435335000 | 0.731979000 |
| 6 | -1.317983000 | 6.142785000 | 1.414391000 | 6 | -1.254703000 | 6.389673000 | 1.325636000 |
| 6 | 1.133426000 | 6.180845000 | -1.400365000 | 6 | 1.087058000 | 6.616464000 | -1.544290000 |
| 6 | 2.311972000 | 5.833671000 | -0.721729000 | 6 | 2.328067000 | 6.389966000 | -0.896865000 |
| 6 | 4.362949000 | 4.502549000 | 0.742910000 | 6 | 4.561626000 | 5.318329000 | 0.512371000 |
| 6 | 5.160555000 | 3.568448000 | 1.420450000 | 6 | 5.509549000 | 4.493413000 | 1.169292000 |
| 6 | -1.314336000 | 6.145377000 | -1.403587000 | 6 | -1.352283000 | 6.370074000 | -1.484282000 |
| 1 | -1.309980000 | 6.132802000 | -2.497300000 | 1 | -1.376522000 | 6.356632000 | -2.577828000 |
| 1 | 1.132308000 | 6.168088000 | -2.494082000 | 1 | 1.059351000 | 6.602497000 | -2.637751000 |
| 1 | -5.255767000 | 3.414553000 | -2.504970000 | 1 | -5.193861000 | 3.424381000 | -2.473973000 |
| 1 | -6.157911000 | 1.144756000 | -2.508263000 | 1 | -6.058884000 | 1.133440000 | -2.444372000 |
| 1 | -5.155847000 | -3.540666000 | -2.511472000 | 1 | -5.132885000 | -3.591200000 | -2.449444000 |
| 1 | -3.403920000 | -5.242794000 | -2.510905000 | 1 | -3.467095000 | -5.386185000 | -2.483219000 |
| 1 | -1.133736000 | -6.144168000 | -2.508899000 | 1 | -1.265795000 | -6.458501000 | -2.532502000 |
| 1 | 1.308555000 | -6.108421000 | -2.505756000 | 1 | 1.174004000 | -6.664183000 | -2.590639000 |
| 1 | 3.551915000 | -5.142649000 | -2.501949000 | 1 | 5.464520000 | -4.482257000 | -2.702266000 |

| | | | | | | | |
|---|---|---|---|---|---|---|---|
| 1 | 5.254490000 | -3.391066000 | -2.498020000 | 1 | 6.736706000 | -2.389904000 | -2.740722000 |
| 1 | 6.121103000 | 1.321241000 | -2.492301000 | 1 | 6.695217000 | 2.424489000 | -2.757943000 |
| 1 | 5.154208000 | 3.564314000 | -2.491386000 | 1 | 5.387368000 | 4.494984000 | -2.734334000 |
| 1 | 3.402367000 | 5.266498000 | -2.492011000 | 1 | 3.420021000 | 5.953067000 | -2.692401000 |
| 1 | -1.140090000 | -6.149252000 | 2.496446000 | 1 | -1.175612000 | -6.432174000 | 2.465950000 |
| 1 | -3.410176000 | -5.247577000 | 2.494474000 | 1 | -3.371815000 | -5.349521000 | 2.515080000 |
| 1 | -5.162050000 | -3.545390000 | 2.493900000 | 1 | -5.028872000 | -3.546385000 | 2.548614000 |
| 1 | -6.164256000 | 1.139998000 | 2.497098000 | 1 | -5.931582000 | 1.183049000 | 2.553095000 |
| 1 | -5.262102000 | 3.409811000 | 2.500424000 | 1 | -5.055885000 | 3.470072000 | 2.523252000 |
| 1 | -1.316431000 | 6.127860000 | 2.508081000 | 1 | -1.225200000 | 6.384969000 | 2.419127000 |
| 1 | 1.125833000 | 6.163358000 | 2.511283000 | 1 | 1.211865000 | 6.619856000 | 2.359207000 |
| 1 | 3.395839000 | 5.261621000 | 2.513368000 | 1 | 3.569387000 | 5.958699000 | 2.304705000 |
| 1 | 5.147672000 | 3.559417000 | 2.513999000 | 1 | 5.529445000 | 4.491086000 | 2.263001000 |
| 1 | 6.114624000 | 1.316383000 | 2.513078000 | 1 | 6.827060000 | 2.414128000 | 2.239667000 |
| 1 | 5.247878000 | -3.395914000 | 2.507359000 | 1 | 6.845902000 | -2.400596000 | 2.257410000 |
| 1 | 3.545423000 | -5.147560000 | 2.503441000 | 1 | 5.564083000 | -4.487354000 | 2.296107000 |
| 1 | 1.302297000 | -6.113936000 | 2.499574000 | 1 | 1.263165000 | -6.648652000 | 2.407940000 |
| 6 | -6.269451000 | -0.079905000 | -0.738444000 | 6 | -6.118675000 | -0.069406000 | -0.667664000 |
| 6 | -6.271324000 | -0.081308000 | 0.724673000 | 6 | -6.083228000 | -0.064489000 | 0.790619000 |
| 6 | -6.137716000 | -1.301585000 | -1.416893000 | 6 | -6.020226000 | -1.312765000 | -1.342068000 |
| 6 | -6.141361000 | -1.303932000 | 1.401391000 | 6 | -5.957787000 | -1.272235000 | 1.468649000 |
| 6 | 6.263359000 | 0.100211000 | -0.722168000 | 6 | 7.087550000 | 1.235595000 | -0.992085000 |
| 6 | 6.261457000 | 0.098794000 | 0.740950000 | 6 | 7.122524000 | 1.241619000 | 0.466215000 |
| 6 | 6.168667000 | -1.124698000 | -1.400909000 | 6 | 7.201744000 | 0.024933000 | -1.666854000 |
| 6 | 6.164987000 | -1.127450000 | 1.417060000 | 6 | 7.269501000 | 0.004782000 | 1.143974000 |
| 1 | -6.122790000 | -1.297605000 | -2.510551000 | 1 | -6.037522000 | -1.315416000 | -2.435843000 |
| 1 | -6.129049000 | -1.302413000 | 2.494822000 | 1 | -5.922176000 | -1.266057000 | 2.561922000 |
| 1 | 6.156399000 | -1.121087000 | -2.494626000 | 1 | 7.166241000 | 0.021155000 | -2.760168000 |
| 1 | 6.149866000 | -1.125967000 | 2.510746000 | 1 | 7.286621000 | 0.008511000 | 2.237728000 |
| 6 | -2.485972000 | 5.763017000 | 0.735031000 | 6 | -2.427171000 | 5.922726000 | 0.679268000 |
| 6 | -2.484013000 | 5.764312000 | -0.728056000 | 6 | -2.462077000 | 5.917295000 | -0.779039000 |
| 6 | -3.566040000 | 5.173817000 | 1.410576000 | 6 | -3.445477000 | 5.288782000 | 1.383095000 |
| 6 | -3.562524000 | 5.176515000 | -1.407500000 | 6 | -3.538142000 | 5.259322000 | -1.427045000 |

| | | | | | | | |
|---|---|---|---|---|---|---|---|
| 1 | -3.559576000 | 5.161436000 | 2.504229000 | 1 | -3.412892000 | 5.285775000 | 2.476444000 |
| 1 | -3.553013000 | 5.166083000 | -2.501125000 | 1 | -3.559232000 | 5.247380000 | -2.520619000 |
| 14 | -0.015156000 | 2.016567000 | -0.284305000 | 6 | 2.443463000 | -6.420269000 | 0.607844000 |
| 14 | -1.863571000 | 0.652784000 | 0.112640000 | 6 | 2.407847000 | -6.425908000 | -0.850508000 |
| 14 | -1.169493000 | -1.508147000 | 0.786028000 | 6 | 3.593847000 | -5.982305000 | 1.254998000 |
| 14 | 1.142103000 | -1.488415000 | 0.785532000 | 6 | 3.553447000 | -5.978700000 | -1.556423000 |
| 14 | 1.842772000 | 0.679114000 | 0.107929000 | 1 | 3.615764000 | -5.970260000 | 2.348620000 |
| 3 | 2.241786000 | 3.245923000 | -0.298683000 | 1 | 3.523180000 | -5.974317000 | -2.649898000 |
| 3 | -2.287226000 | 3.210245000 | -0.343226000 | 14 | 0.442105000 | 1.975825000 | 0.628022000 |
| 3 | -3.703380000 | -1.090378000 | 0.659247000 | 14 | -1.371163000 | 0.485124000 | 0.681638000 |
| 3 | -0.004031000 | -3.833197000 | 0.882627000 | 14 | -0.510875000 | -1.698385000 | 0.670132000 |
| 3 | 3.677716000 | -1.054088000 | 0.670287000 | 14 | 1.834901000 | -1.565782000 | 0.609276000 |
| 3 | -0.014325000 | 0.558591000 | 1.964651000 | 14 | 2.419511000 | 0.707379000 | 0.583170000 |
| 3 | -0.008483000 | -0.425432000 | -1.355829000 | 3 | 2.679946000 | 3.265397000 | 0.557025000 |
| | | | | 3 | -1.933538000 | 2.998891000 | 0.669720000 |
| | | | | 3 | -3.076779000 | -1.449873000 | 0.728645000 |
| | | | | 3 | 0.771662000 | -3.923711000 | 0.637630000 |
| | | | | 3 | 4.355162000 | -0.984043000 | 0.539175000 |
| | | | | 3 | 0.606549000 | -0.011024000 | 2.324612000 |
| | | | | 3 | 0.519702000 | -0.027439000 | -1.051910000 |
| $Si_5Li_7^+$@[18]cyclacene | | | | $Si_5Li_7^+$@[19]cyclacene | | | |
| 6 | -6.241007000 | 2.988121000 | 1.388901000 | 6 | -7.422982000 | 1.965443000 | 1.158022000 |
| 6 | -5.737741000 | 4.073550000 | 0.672752000 | 6 | -7.071549000 | 3.120606000 | 0.434550000 |
| 6 | -5.022831000 | 5.115648000 | 1.304988000 | 6 | -6.538967000 | 4.249263000 | 1.066197000 |
| 6 | -5.779647000 | 4.040415000 | -0.785601000 | 6 | -7.087944000 | 3.068023000 | -1.028176000 |
| 6 | -5.117174000 | 5.034753000 | -1.504308000 | 6 | -6.565324000 | 4.156844000 | -1.751184000 |
| 6 | -4.232121000 | 5.938124000 | -0.873776000 | 6 | -5.856916000 | 5.183214000 | -1.117823000 |
| 6 | -4.190429000 | 5.971444000 | 0.584561000 | 6 | -5.840575000 | 5.235818000 | 0.344903000 |
| 6 | -2.420464000 | -6.364334000 | 0.815620000 | 6 | -2.601420000 | -7.000447000 | 0.748213000 |
| 6 | -6.329515000 | 2.903989000 | -1.420478000 | 6 | -7.452354000 | 1.874191000 | -1.659395000 |
| 1 | -5.142222000 | 5.004064000 | -2.597457000 | 1 | -6.567263000 | 4.111778000 | -2.843976000 |

| | | | | | | | |
|---|---|---|---|---|---|---|---|
| 6 | -3.278504000 | 6.656017000 | -1.594482000 | 6 | -5.016300000 | 6.053937000 | -1.836571000 |
| 6 | -3.179872000 | 6.731939000 | 1.214796000 | 6 | -4.992501000 | 6.148334000 | 0.980804000 |
| 6 | -2.110520000 | 7.263705000 | 0.495057000 | 6 | -4.016088000 | 6.867420000 | 0.266031000 |
| 6 | -2.152047000 | 7.230139000 | -0.963280000 | 6 | -4.032420000 | 6.814858000 | -1.196700000 |
| 1 | -3.305595000 | 6.623395000 | -2.687530000 | 1 | -5.020565000 | 6.006060000 | -2.929230000 |
| 1 | -3.143725000 | 6.750031000 | 2.307917000 | 1 | -4.972673000 | 6.178761000 | 2.073920000 |
| 6 | -2.462620000 | -6.397317000 | -0.642702000 | 6 | -2.617476000 | -7.052955000 | -0.714522000 |
| 1 | -6.201396000 | 3.010475000 | 2.481824000 | 1 | -7.399158000 | 2.001962000 | 2.250874000 |
| 1 | -4.984505000 | 5.135600000 | 2.398002000 | 1 | -6.516629000 | 4.282396000 | 2.159187000 |
| 6 | -1.260685000 | -6.780046000 | 1.468541000 | 6 | -1.413443000 | -7.334950000 | 1.425284000 |
| 1 | -6.353264000 | 2.875791000 | -2.513724000 | 1 | -7.452884000 | 1.832601000 | -2.752325000 |
| 6 | -1.320993000 | -6.849526000 | -1.341993000 | 6 | -1.454494000 | -7.434114000 | -1.391791000 |
| 6 | -0.060348000 | -7.020791000 | 0.762774000 | 6 | -0.208102000 | -7.517476000 | 0.740186000 |
| 6 | -0.102557000 | -7.053914000 | -0.695549000 | 6 | -0.223599000 | -7.569938000 | -0.722416000 |
| 1 | -1.227287000 | -6.746897000 | 2.561395000 | 1 | -1.398778000 | -7.284223000 | 2.517645000 |
| 1 | -1.349822000 | -6.866192000 | -2.435354000 | 1 | -1.464228000 | -7.461720000 | -2.485121000 |
| 6 | 1.177383000 | -7.042627000 | 1.404505000 | 6 | 1.033326000 | -7.470035000 | 1.403016000 |
| 6 | 1.117767000 | -7.105287000 | -1.406203000 | 6 | 0.992153000 | -7.572286000 | -1.413887000 |
| 6 | -0.911612000 | 7.661785000 | 1.128256000 | 6 | -2.934324000 | 7.476727000 | 0.910260000 |
| 6 | -1.012813000 | 7.592201000 | -1.681090000 | 6 | -2.956430000 | 7.379737000 | -1.907041000 |
| 6 | 0.276694000 | 7.810093000 | 0.413976000 | 6 | -1.776072000 | 7.855341000 | 0.205690000 |
| 6 | 0.235172000 | 7.776515000 | -1.044353000 | 6 | -1.792435000 | 7.802891000 | -1.257039000 |
| 6 | 2.342269000 | -6.908191000 | -0.769076000 | 6 | 2.212444000 | -7.322421000 | -0.758282000 |
| 6 | 2.384495000 | -6.874940000 | 0.689246000 | 6 | 2.228523000 | -7.269821000 | 0.704450000 |
| 6 | 3.568053000 | -6.499067000 | 1.323432000 | 6 | 3.401840000 | -6.842572000 | 1.354185000 |
| 6 | 3.506817000 | -6.554927000 | -1.487373000 | 6 | 3.361419000 | -6.947967000 | -1.462615000 |
| 6 | 4.648974000 | -5.942928000 | 0.602940000 | 6 | 4.468562000 | -6.281925000 | 0.644061000 |
| 6 | 4.606772000 | -5.976273000 | -0.855402000 | 6 | 4.452449000 | -6.334476000 | -0.818667000 |
| 6 | 7.203945000 | -3.310664000 | 1.146305000 | 6 | 7.006741000 | -3.615320000 | 1.198115000 |
| 6 | 7.133739000 | -3.356346000 | -1.664505000 | 6 | 6.970219000 | -3.725120000 | -1.618533000 |
| 6 | 1.536135000 | 7.804178000 | 1.054777000 | 6 | -0.565857000 | 8.104056000 | 0.861461000 |
| 6 | 8.159548000 | 0.207216000 | 0.361919000 | 6 | 8.041223000 | -0.143272000 | 0.383795000 |
| 6 | 8.055654000 | -1.011573000 | 1.069445000 | 6 | 7.890842000 | -1.331537000 | 1.106229000 |

| | | | | | | | |
|---|---|---|---|---|---|---|---|
| 6 | 1.434506000 | 7.741762000 | -1.754729000 | 6 | -0.587156000 | 8.004209000 | -1.955739000 |
| 6 | 8.117826000 | 0.173618000 | -1.096416000 | 6 | 8.024882000 | -0.195663000 | -1.078940000 |
| 6 | 7.979071000 | -1.054888000 | -1.741204000 | 6 | 7.857151000 | -1.442438000 | -1.710425000 |
| 1 | 7.227814000 | -3.281792000 | 2.239528000 | 1 | 7.008840000 | -3.569968000 | 2.290895000 |
| 1 | 3.595945000 | -6.466383000 | 2.416458000 | 1 | 3.409377000 | -6.792505000 | 2.446731000 |
| 1 | 1.207984000 | -7.009221000 | 2.497433000 | 1 | 1.044397000 | -7.418905000 | 2.495453000 |
| 1 | 1.086135000 | -7.121569000 | -2.499492000 | 1 | 0.978716000 | -7.599900000 | -2.507177000 |
| 1 | 3.472341000 | -6.571749000 | -2.580568000 | 1 | 3.344444000 | -6.976595000 | -2.555826000 |
| 1 | 7.095106000 | -3.376808000 | -2.757497000 | 1 | 6.947633000 | -3.758540000 | -2.711510000 |
| 1 | 8.078540000 | -0.985331000 | 2.162757000 | 1 | 7.891498000 | -1.289618000 | 2.199147000 |
| 1 | 1.566868000 | 7.820782000 | 2.148086000 | 1 | -0.552696000 | 8.131460000 | 1.954759000 |
| 1 | -0.878033000 | 7.678641000 | 2.221479000 | 1 | -2.917569000 | 7.505097000 | 2.003482000 |
| 1 | -1.042512000 | 7.558480000 | -2.774035000 | 1 | -2.963692000 | 7.329885000 | -2.999598000 |
| 1 | 1.401966000 | 7.708121000 | -2.847595000 | 1 | -0.597933000 | 7.953329000 | -3.048224000 |
| 1 | 7.939435000 | -1.077924000 | -2.834109000 | 1 | 7.833233000 | -1.479306000 | -2.803264000 |
| 6 | 7.659712000 | -2.232534000 | -1.028433000 | 6 | 7.507916000 | -2.587673000 | -0.987305000 |
| 6 | 7.701673000 | -2.198975000 | 0.429902000 | 6 | 7.524258000 | -2.535306000 | 0.475443000 |
| 6 | -6.627126000 | 1.742642000 | -0.708537000 | 6 | -7.605012000 | 0.675999000 | -0.936596000 |
| 6 | -6.585013000 | 1.775659000 | 0.749813000 | 6 | -7.588561000 | 0.728629000 | 0.526130000 |
| 6 | -3.544963000 | -5.815385000 | -1.301612000 | 6 | -3.739076000 | -6.547272000 | -1.398349000 |
| 6 | -3.481702000 | -5.739560000 | 1.508676000 | 6 | -3.699204000 | -6.451198000 | 1.418759000 |
| 6 | 8.115006000 | 1.440025000 | 1.011861000 | 6 | 8.025567000 | 1.113796000 | 1.016947000 |
| 6 | 8.031727000 | 1.396854000 | -1.798604000 | 6 | 7.995122000 | 1.002675000 | -1.799740000 |
| 6 | 2.670887000 | 7.517604000 | -1.108245000 | 6 | 0.643710000 | 8.050546000 | -1.292951000 |
| 6 | 2.712424000 | 7.551214000 | 0.350084000 | 6 | 0.660071000 | 8.102867000 | 0.169787000 |
| 6 | -4.481976000 | -5.009722000 | -0.616239000 | 6 | -4.734674000 | -5.822285000 | -0.735241000 |
| 6 | -4.439849000 | -4.976776000 | 0.842100000 | 6 | -4.718518000 | -5.769668000 | 0.727484000 |
| 6 | 7.770197000 | 2.598702000 | -1.141762000 | 6 | 7.776823000 | 2.239205000 | -1.163584000 |
| 6 | 7.811753000 | 2.632299000 | 0.316581000 | 6 | 7.793198000 | 2.291627000 | 0.299145000 |
| 6 | 7.285578000 | 3.733339000 | -1.830595000 | 6 | 7.369899000 | 3.370897000 | -1.877828000 |
| 6 | 7.375597000 | 3.778741000 | 0.979630000 | 6 | 7.397368000 | 3.481308000 | 0.938923000 |
| 6 | -6.432853000 | -1.932858000 | -1.307319000 | 6 | -6.965329000 | -2.939169000 | -1.492408000 |

| | | | | | | | |
|---|---|---|---|---|---|---|---|
| 6 | -6.358219000 | -1.848403000 | 1.503089000 | 6 | -6.930030000 | -2.845701000 | 1.324833000 |
| 6 | -6.784473000 | 0.494580000 | -1.352232000 | 6 | -7.590364000 | -0.571057000 | -1.570276000 |
| 6 | -6.702724000 | 0.579886000 | 1.457318000 | 6 | -7.557971000 | -0.479839000 | 1.247207000 |
| 6 | -6.683151000 | -0.706347000 | -0.651079000 | 6 | -7.357168000 | -1.759740000 | -0.850274000 |
| 6 | -6.641001000 | -0.673307000 | 0.807364000 | 6 | -7.340896000 | -1.707121000 | 0.609792000 |
| 6 | 3.798099000 | 7.087837000 | -1.807513000 | 6 | 1.859512000 | 7.866056000 | -1.977898000 |
| 6 | 3.897909000 | 7.143487000 | 1.002208000 | 6 | 1.880982000 | 7.969085000 | 0.839132000 |
| 6 | 6.654203000 | 4.780007000 | -1.159679000 | 6 | 6.787669000 | 4.478425000 | -1.233023000 |
| 6 | 6.695789000 | 4.813659000 | 0.298651000 | 6 | 6.804098000 | 4.530887000 | 0.229724000 |
| 1 | 3.762830000 | 7.055064000 | -2.900319000 | 1 | 1.845038000 | 7.815225000 | -3.070336000 |
| 1 | 3.925928000 | 7.160722000 | 2.095579000 | 1 | 1.890467000 | 7.996609000 | 1.932457000 |
| 1 | 7.399403000 | 3.799587000 | 2.073038000 | 1 | 7.398796000 | 3.515983000 | 2.032092000 |
| 1 | 7.246558000 | 3.704733000 | -2.923391000 | 1 | 7.346991000 | 3.326706000 | -2.970414000 |
| 1 | 7.991990000 | 1.371020000 | -2.891444000 | 1 | 7.971200000 | 0.962073000 | -2.892445000 |
| 1 | 8.137858000 | 1.463456000 | 2.105238000 | 1 | 8.026050000 | 1.152008000 | 2.110000000 |
| 1 | -6.807608000 | 0.469118000 | -2.445587000 | 1 | -7.590632000 | -0.608806000 | -2.663205000 |
| 1 | -6.662633000 | 0.604936000 | 2.550139000 | 1 | -7.533917000 | -0.439586000 | 2.339959000 |
| 1 | -6.318844000 | -1.820564000 | 2.595581000 | 1 | -6.906727000 | -2.804129000 | 2.417866000 |
| 1 | -6.456323000 | -1.955203000 | -2.400386000 | 1 | -6.966657000 | -2.973632000 | -2.585264000 |
| 1 | -3.571281000 | -5.833340000 | -2.395016000 | 1 | -3.745402000 | -6.576241000 | -2.491668000 |
| 1 | -3.445691000 | -5.707529000 | 2.601482000 | 1 | -3.681057000 | -6.401848000 | 2.511215000 |
| 6 | -5.941416000 | -3.041517000 | -0.619006000 | 6 | -6.368117000 | -3.998163000 | -0.782585000 |
| 6 | -5.899334000 | -3.008539000 | 0.839235000 | 6 | -6.351256000 | -3.944841000 | 0.680316000 |
| 6 | -5.312809000 | -4.114853000 | -1.289549000 | 6 | -5.638069000 | -4.998622000 | -1.432889000 |
| 6 | -5.244588000 | -4.033889000 | 1.520540000 | 6 | -5.600194000 | -4.905101000 | 1.384280000 |
| 6 | 5.821583000 | 5.701481000 | -1.833738000 | 6 | 6.042731000 | 5.430209000 | -1.937038000 |
| 6 | 5.917367000 | 5.751271000 | 0.976245000 | 6 | 6.067497000 | 5.538891000 | 0.879882000 |
| 6 | 4.890835000 | 6.481240000 | -1.148160000 | 6 | 5.154433000 | 6.302559000 | -1.280308000 |
| 6 | 4.932417000 | 6.514920000 | 0.310177000 | 6 | 5.170843000 | 6.355034000 | 0.182418000 |
| 1 | -5.337188000 | -4.134895000 | -2.382913000 | 1 | -5.641632000 | -5.029997000 | -2.526150000 |
| 1 | -5.206474000 | -4.003687000 | 2.613328000 | 1 | -5.579580000 | -4.858384000 | 2.476909000 |
| 1 | 5.784137000 | 5.670480000 | -2.926524000 | 1 | 6.021875000 | 5.383014000 | -3.029536000 |
| 1 | 5.943029000 | 5.770040000 | 2.069649000 | 1 | 6.070890000 | 5.570459000 | 1.973136000 |

| | | | | | | | |
|---|---|---|---|---|---|---|---|
| 6 | 5.652157000 | -5.208393000 | 1.234125000 | 6 | 5.460095000 | -5.514278000 | 1.283553000 |
| 6 | 6.487644000 | -4.325762000 | 0.513215000 | 6 | 6.293196000 | -4.650475000 | 0.565164000 |
| 6 | 5.587259000 | -5.258422000 | -1.576733000 | 6 | 5.421316000 | -5.622229000 | -1.533127000 |
| 6 | 6.445596000 | -4.359269000 | -0.945124000 | 6 | 6.277022000 | -4.702927000 | -0.897555000 |
| 1 | 5.550428000 | -5.276832000 | -2.669827000 | 1 | 5.401242000 | -5.652835000 | -2.626233000 |
| 1 | 5.677786000 | -5.177260000 | 2.327246000 | 1 | 5.464518000 | -5.466136000 | 2.376202000 |
| 14 | 0.831702000 | 0.791560000 | -0.682740000 | 6 | 4.166611000 | 7.085258000 | 0.845430000 |
| 14 | -1.375587000 | -0.012832000 | -0.451071000 | 6 | 3.053871000 | 7.585947000 | 0.161670000 |
| 14 | -1.235722000 | -2.318999000 | -0.246046000 | 6 | 4.143943000 | 6.979035000 | -1.971401000 |
| 14 | 1.015185000 | -2.953734000 | -0.328137000 | 6 | 3.037445000 | 7.533475000 | -1.300873000 |
| 14 | 2.310077000 | -1.046125000 | -0.587536000 | 1 | 4.172575000 | 7.114166000 | 1.938749000 |
| 3 | 3.290161000 | 1.221484000 | -0.718569000 | 1 | 4.125986000 | 6.929613000 | -3.063854000 |
| 3 | -1.038667000 | 2.436551000 | -0.549122000 | 14 | 0.473465000 | 0.243944000 | 0.106963000 |
| 3 | -3.609580000 | -1.510841000 | -0.307015000 | 14 | -1.730939000 | -0.598487000 | -0.006601000 |
| 3 | -0.716386000 | -4.775099000 | -0.059572000 | 14 | -1.557815000 | -2.915340000 | 0.038021000 |
| 3 | 3.460414000 | -3.476939000 | -0.606327000 | 14 | 0.696114000 | -3.509278000 | 0.147274000 |
| 3 | 0.418326000 | -0.942079000 | 1.221976000 | 14 | 1.969040000 | -1.573562000 | 0.184882000 |
| 3 | 0.205666000 | -1.253628000 | -2.144035000 | 3 | 2.927990000 | 0.695181000 | 0.128395000 |
| | | | | 3 | -1.413923000 | 1.844500000 | -0.002144000 |
| | | | | 3 | -3.914878000 | -2.142001000 | -0.002091000 |
| | | | | 3 | -1.006109000 | -5.365660000 | -0.051645000 |
| | | | | 3 | 3.149843000 | -3.997996000 | 0.374692000 |
| | | | | 3 | -0.115800000 | -1.640577000 | 1.793470000 |
| | | | | 3 | 0.045982000 | -1.674861000 | -1.602844000 |
| $Si_5Li_7^+$@[20]cyclacene (A-structure) | | | | $Si_5Li_7^+$@[20]cyclacene (B-structure) | | | |
| 6 | -7.422982000 | 1.965443000 | 1.158022000 | 6 | -7.422982000 | 1.965443000 | 1.158022000 |
| 6 | -7.071549000 | 3.120606000 | 0.434550000 | 6 | -7.071549000 | 3.120606000 | 0.434550000 |
| 6 | -6.538967000 | 4.249263000 | 1.066197000 | 6 | -6.538967000 | 4.249263000 | 1.066197000 |
| 6 | -7.087944000 | 3.068023000 | -1.028176000 | 6 | -7.087944000 | 3.068023000 | -1.028176000 |
| 6 | -6.565324000 | 4.156844000 | -1.751184000 | 6 | -6.565324000 | 4.156844000 | -1.751184000 |
| 6 | -5.856916000 | 5.183214000 | -1.117823000 | 6 | -5.856916000 | 5.183214000 | -1.117823000 |

| | | | | | | | |
|---|---|---|---|---|---|---|---|
| 6 | -5.840575000 | 5.235818000 | 0.344903000 | 6 | -5.840575000 | 5.235818000 | 0.344903000 |
| 6 | -2.600999000 | -7.000281000 | 0.749119000 | 6 | -2.601420000 | -7.000447000 | 0.748213000 |
| 6 | -7.452354000 | 1.874191000 | -1.659395000 | 6 | -7.452354000 | 1.874191000 | -1.659395000 |
| 1 | -6.567263000 | 4.111778000 | -2.843976000 | 1 | -6.567263000 | 4.111778000 | -2.843976000 |
| 6 | -5.016300000 | 6.053937000 | -1.836571000 | 6 | -5.016300000 | 6.053937000 | -1.836571000 |
| 6 | -4.992501000 | 6.148334000 | 0.980804000 | 6 | -4.992501000 | 6.148334000 | 0.980804000 |
| 6 | -4.016088000 | 6.867420000 | 0.266031000 | 6 | -4.016088000 | 6.867420000 | 0.266031000 |
| 6 | -4.032420000 | 6.814858000 | -1.196700000 | 6 | -4.032420000 | 6.814858000 | -1.196700000 |
| 1 | -5.020565000 | 6.006060000 | -2.929230000 | 1 | -5.020565000 | 6.006060000 | -2.929230000 |
| 1 | -4.972673000 | 6.178761000 | 2.073920000 | 1 | -4.972673000 | 6.178761000 | 2.073920000 |
| 6 | -2.618560000 | -7.052104000 | -0.713879000 | 6 | -2.617476000 | -7.052955000 | -0.714522000 |
| 1 | -7.399158000 | 2.001962000 | 2.250874000 | 1 | -7.399158000 | 2.001962000 | 2.250874000 |
| 1 | -6.516629000 | 4.282396000 | 2.159187000 | 1 | -6.516629000 | 4.282396000 | 2.159187000 |
| 6 | -1.412491000 | -7.335244000 | 1.425594000 | 6 | -1.413443000 | -7.334950000 | 1.425284000 |
| 1 | -7.452884000 | 1.832601000 | -2.752325000 | 1 | -7.452884000 | 1.832601000 | -2.752325000 |
| 6 | -1.453479000 | -7.434556000 | -1.394262000 | 6 | -1.454494000 | -7.434114000 | -1.391791000 |
| 6 | -0.206792000 | -7.517693000 | 0.740355000 | 6 | -0.208102000 | -7.517476000 | 0.740186000 |
| 6 | -0.225254000 | -7.569720000 | -0.721299000 | 6 | -0.223599000 | -7.569938000 | -0.722416000 |
| 1 | -1.399258000 | -7.284124000 | 2.517646000 | 1 | -1.398778000 | -7.284223000 | 2.517645000 |
| 1 | -1.464490000 | -7.461779000 | -2.485117000 | 1 | -1.464228000 | -7.461720000 | -2.485121000 |
| 6 | 1.033254000 | -7.469819000 | 1.403135000 | 6 | 1.033326000 | -7.470035000 | 1.403016000 |
| 6 | 0.992146000 | -7.572316000 | -1.413899000 | 6 | 0.992153000 | -7.572286000 | -1.413887000 |
| 6 | -2.934324000 | 7.476727000 | 0.910260000 | 6 | -2.934324000 | 7.476727000 | 0.910260000 |
| 6 | -2.956430000 | 7.379737000 | -1.907041000 | 6 | -2.956430000 | 7.379737000 | -1.907041000 |
| 6 | -1.776072000 | 7.855341000 | 0.205690000 | 6 | -1.776072000 | 7.855341000 | 0.205690000 |
| 6 | -1.792435000 | 7.802891000 | -1.257039000 | 6 | -1.792435000 | 7.802891000 | -1.257039000 |
| 6 | 2.212444000 | -7.322421000 | -0.758282000 | 6 | 2.212444000 | -7.322421000 | -0.758282000 |
| 6 | 2.228531000 | -7.269903000 | 0.704441000 | 6 | 2.228523000 | -7.269821000 | 0.704450000 |
| 6 | 3.401840000 | -6.842572000 | 1.354185000 | 6 | 3.401840000 | -6.842572000 | 1.354185000 |
| 6 | 3.361419000 | -6.947967000 | -1.462615000 | 6 | 3.361419000 | -6.947967000 | -1.462615000 |
| 6 | 4.468562000 | -6.281925000 | 0.644061000 | 6 | 4.468562000 | -6.281925000 | 0.644061000 |
| 6 | 4.452449000 | -6.334476000 | -0.818667000 | 6 | 4.452449000 | -6.334476000 | -0.818667000 |
| 6 | 7.006741000 | -3.615320000 | 1.198115000 | 6 | 7.006741000 | -3.615320000 | 1.198115000 |

| | | | | | | | |
|---|---|---|---|---|---|---|---|
| 6 | 6.970219000 | -3.725120000 | -1.618533000 | 6 | 6.970219000 | -3.725120000 | -1.618533000 |
| 6 | -0.565857000 | 8.104056000 | 0.861461000 | 6 | -0.565857000 | 8.104056000 | 0.861461000 |
| 6 | 8.041223000 | -0.143272000 | 0.383795000 | 6 | 8.041223000 | -0.143272000 | 0.383795000 |
| 6 | 7.890842000 | -1.331537000 | 1.106229000 | 6 | 7.890842000 | -1.331537000 | 1.106229000 |
| 6 | -0.587156000 | 8.004209000 | -1.955739000 | 6 | -0.587156000 | 8.004209000 | -1.955739000 |
| 6 | 8.024882000 | -0.195663000 | -1.078940000 | 6 | 8.024882000 | -0.195663000 | -1.078940000 |
| 6 | 7.857151000 | -1.442438000 | -1.710425000 | 6 | 7.857151000 | -1.442438000 | -1.710425000 |
| 1 | 7.008840000 | -3.569968000 | 2.290895000 | 1 | 7.008840000 | -3.569968000 | 2.290895000 |
| 1 | 3.409377000 | -6.792505000 | 2.446731000 | 1 | 3.409377000 | -6.792505000 | 2.446731000 |
| 1 | 1.044398000 | -7.418952000 | 2.495455000 | 1 | 1.044397000 | -7.418905000 | 2.495453000 |
| 1 | 0.978716000 | -7.599900000 | -2.507177000 | 1 | 0.978716000 | -7.599900000 | -2.507177000 |
| 1 | 3.344444000 | -6.976595000 | -2.555826000 | 1 | 3.344444000 | -6.976595000 | -2.555826000 |
| 1 | 6.947633000 | -3.758540000 | -2.711510000 | 1 | 6.947633000 | -3.758540000 | -2.711510000 |
| 1 | 7.891498000 | -1.289618000 | 2.199147000 | 1 | 7.891498000 | -1.289618000 | 2.199147000 |
| 1 | -0.552696000 | 8.131460000 | 1.954759000 | 1 | -0.552696000 | 8.131460000 | 1.954759000 |
| 1 | -2.917569000 | 7.505097000 | 2.003482000 | 1 | -2.917569000 | 7.505097000 | 2.003482000 |
| 1 | -2.963692000 | 7.329885000 | -2.999598000 | 1 | -2.963692000 | 7.329885000 | -2.999598000 |
| 1 | -0.597933000 | 7.953329000 | -3.048224000 | 1 | -0.597933000 | 7.953329000 | -3.048224000 |
| 1 | 7.833233000 | -1.479306000 | -2.803264000 | 1 | 7.833233000 | -1.479306000 | -2.803264000 |
| 6 | 7.507916000 | -2.587673000 | -0.987305000 | 6 | 7.507916000 | -2.587673000 | -0.987305000 |
| 6 | 7.524258000 | -2.535306000 | 0.475443000 | 6 | 7.524258000 | -2.535306000 | 0.475443000 |
| 6 | -7.605012000 | 0.675999000 | -0.936596000 | 6 | -7.605012000 | 0.675999000 | -0.936596000 |
| 6 | -7.588561000 | 0.728629000 | 0.526130000 | 6 | -7.588561000 | 0.728629000 | 0.526130000 |
| 6 | -3.738924000 | -6.547526000 | -1.398784000 | 6 | -3.739076000 | -6.547272000 | -1.398349000 |
| 6 | -3.699489000 | -6.451302000 | 1.418376000 | 6 | -3.699204000 | -6.451198000 | 1.418759000 |
| 6 | 8.025567000 | 1.113796000 | 1.016947000 | 6 | 8.025567000 | 1.113796000 | 1.016947000 |
| 6 | 7.995122000 | 1.002675000 | -1.799740000 | 6 | 7.995122000 | 1.002675000 | -1.799740000 |
| 6 | 0.643710000 | 8.050546000 | -1.292951000 | 6 | 0.643710000 | 8.050546000 | -1.292951000 |
| 6 | 0.660071000 | 8.102867000 | 0.169787000 | 6 | 0.660071000 | 8.102867000 | 0.169787000 |
| 6 | -4.734743000 | -5.822404000 | -0.735215000 | 6 | -4.734674000 | -5.822285000 | -0.735241000 |
| 6 | -4.718470000 | -5.769586000 | 0.727495000 | 6 | -4.718518000 | -5.769668000 | 0.727484000 |
| 6 | 7.776823000 | 2.239205000 | -1.163584000 | 6 | 7.776823000 | 2.239205000 | -1.163584000 |

| | | | | | | | |
|---|---|---|---|---|---|---|---|
| 6 | 7.793198000 | 2.291627000 | 0.299145000 | 6 | 7.793198000 | 2.291627000 | 0.299145000 |
| 6 | 7.369899000 | 3.370897000 | -1.877828000 | 6 | 7.369899000 | 3.370897000 | -1.877828000 |
| 6 | 7.397368000 | 3.481308000 | 0.938923000 | 6 | 7.397368000 | 3.481308000 | 0.938923000 |
| 6 | -6.965329000 | -2.939169000 | -1.492408000 | 6 | -6.965329000 | -2.939169000 | -1.492408000 |
| 6 | -6.930030000 | -2.845701000 | 1.324833000 | 6 | -6.930030000 | -2.845701000 | 1.324833000 |
| 6 | -7.590364000 | -0.571057000 | -1.570276000 | 6 | -7.590364000 | -0.571057000 | -1.570276000 |
| 6 | -7.557971000 | -0.479839000 | 1.247207000 | 6 | -7.557971000 | -0.479839000 | 1.247207000 |
| 6 | -7.357168000 | -1.759740000 | -0.850274000 | 6 | -7.357168000 | -1.759740000 | -0.850274000 |
| 6 | -7.340896000 | -1.707121000 | 0.609792000 | 6 | -7.340896000 | -1.707121000 | 0.609792000 |
| 6 | 1.859512000 | 7.866056000 | -1.977898000 | 6 | 1.859512000 | 7.866056000 | -1.977898000 |
| 6 | 1.880982000 | 7.969085000 | 0.839132000 | 6 | 1.880982000 | 7.969085000 | 0.839132000 |
| 6 | 6.787669000 | 4.478425000 | -1.233023000 | 6 | 6.787669000 | 4.478425000 | -1.233023000 |
| 6 | 6.804098000 | 4.530887000 | 0.229724000 | 6 | 6.804098000 | 4.530887000 | 0.229724000 |
| 1 | 1.845038000 | 7.815225000 | -3.070336000 | 1 | 1.845038000 | 7.815225000 | -3.070336000 |
| 1 | 1.890467000 | 7.996609000 | 1.932457000 | 1 | 1.890467000 | 7.996609000 | 1.932457000 |
| 1 | 7.398796000 | 3.515983000 | 2.032092000 | 1 | 7.398796000 | 3.515983000 | 2.032092000 |
| 1 | 7.346991000 | 3.326706000 | -2.970414000 | 1 | 7.346991000 | 3.326706000 | -2.970414000 |
| 1 | 7.971200000 | 0.962073000 | -2.892445000 | 1 | 7.971200000 | 0.962073000 | -2.892445000 |
| 1 | 8.026050000 | 1.152008000 | 2.110000000 | 1 | 8.026050000 | 1.152008000 | 2.110000000 |
| 1 | -7.590632000 | -0.608806000 | -2.663205000 | 1 | -7.590632000 | -0.608806000 | -2.663205000 |
| 1 | -7.533917000 | -0.439586000 | 2.339959000 | 1 | -7.533917000 | -0.439586000 | 2.339959000 |
| 1 | -6.906727000 | -2.804129000 | 2.417866000 | 1 | -6.906727000 | -2.804129000 | 2.417866000 |
| 1 | -6.966657000 | -2.973632000 | -2.585264000 | 1 | -6.966657000 | -2.973632000 | -2.585264000 |
| 1 | -3.745350000 | -6.576130000 | -2.491671000 | 1 | -3.745402000 | -6.576241000 | -2.491668000 |
| 1 | -3.681102000 | -6.401944000 | 2.511220000 | 1 | -3.681057000 | -6.401848000 | 2.511215000 |
| 6 | -6.368117000 | -3.998163000 | -0.782585000 | 6 | -6.368117000 | -3.998163000 | -0.782585000 |
| 6 | -6.351256000 | -3.944841000 | 0.680316000 | 6 | -6.351256000 | -3.944841000 | 0.680316000 |
| 6 | -5.638069000 | -4.998622000 | -1.432889000 | 6 | -5.638069000 | -4.998622000 | -1.432889000 |
| 6 | -5.600194000 | -4.905101000 | 1.384280000 | 6 | -5.600194000 | -4.905101000 | 1.384280000 |
| 6 | 6.042731000 | 5.430209000 | -1.937038000 | 6 | 6.042731000 | 5.430209000 | -1.937038000 |
| 6 | 6.067497000 | 5.538891000 | 0.879882000 | 6 | 6.067497000 | 5.538891000 | 0.879882000 |
| 6 | 5.154433000 | 6.302559000 | -1.280308000 | 6 | 5.154433000 | 6.302559000 | -1.280308000 |
| 6 | 5.170843000 | 6.355034000 | 0.182418000 | 6 | 5.170843000 | 6.355034000 | 0.182418000 |

| | |
|---|---|
| 1 -5.641632000 -5.029997000 -2.526150000 | 1 -5.641632000 -5.029997000 -2.526150000 |
| 1 -5.579580000 -4.858384000 2.476909000 | 1 -5.579580000 -4.858384000 2.476909000 |
| 1 6.021875000 5.383014000 -3.029536000 | 1 6.021875000 5.383014000 -3.029536000 |
| 1 6.070890000 5.570459000 1.973136000 | 1 6.070890000 5.570459000 1.973136000 |
| 6 5.460095000 -5.514278000 1.283553000 | 6 5.460095000 -5.514278000 1.283553000 |
| 6 6.293196000 -4.650475000 0.565164000 | 6 6.293196000 -4.650475000 0.565164000 |
| 6 5.421316000 -5.622229000 -1.533127000 | 6 5.421316000 -5.622229000 -1.533127000 |
| 6 6.277022000 -4.702927000 -0.897555000 | 6 6.277022000 -4.702927000 -0.897555000 |
| 1 5.401242000 -5.652835000 -2.626233000 | 1 5.401242000 -5.652835000 -2.626233000 |
| 1 5.464518000 -5.466136000 2.376202000 | 1 5.464518000 -5.466136000 2.376202000 |
| 6 4.166611000 7.085258000 0.845430000 | 6 4.166611000 7.085258000 0.845430000 |
| 6 3.053871000 7.585947000 0.161670000 | 6 3.053871000 7.585947000 0.161670000 |
| 6 4.143943000 6.979035000 -1.971401000 | 6 4.143943000 6.979035000 -1.971401000 |
| 6 3.037445000 7.533475000 -1.300873000 | 6 3.037445000 7.533475000 -1.300873000 |
| 1 4.172575000 7.114166000 1.938749000 | 1 4.172575000 7.114166000 1.938749000 |
| 1 4.125986000 6.929613000 -3.063854000 | 1 4.125986000 6.929613000 -3.063854000 |
| 14 0.009377000 -3.246739000 3.931221000 | 14 0.473465000 0.243944000 0.106963000 |
| 14 -1.873403000 -3.148920000 2.554624000 | 14 -1.730939000 -0.598487000 -0.006601000 |
| 14 -1.164593000 -3.229815000 0.318841000 | 14 -1.557815000 -2.915340000 0.038021000 |
| 14 1.162496000 -3.405844000 0.312628000 | 14 0.696114000 -3.509278000 0.147274000 |
| 14 1.880408000 -3.422455000 2.547551000 | 14 1.969040000 -1.573562000 0.184882000 |
| 3 2.247153000 -3.078284000 4.953065000 | 3 2.927990000 0.695181000 0.128395000 |
| 3 -2.177976000 -2.772014000 4.963314000 | 3 -1.413923000 1.844500000 -0.002144000 |
| 3 -3.621171000 -3.650421000 0.764407000 | 3 -3.914878000 -2.142001000 -0.002091000 |
| 3 -0.156368000 -5.153201000 -1.103276000 | 3 -1.006109000 -5.365660000 -0.051645000 |
| 3 3.559653000 -4.094793000 0.757584000 | 3 3.149843000 -3.997996000 0.374692000 |
| 3 -0.120995000 -5.014062000 1.999481000 | 3 -0.115800000 -1.640577000 1.793470000 |
| 3 0.125315000 -1.605753000 1.867866000 | 3 0.045982000 -1.674861000 -1.602844000 |

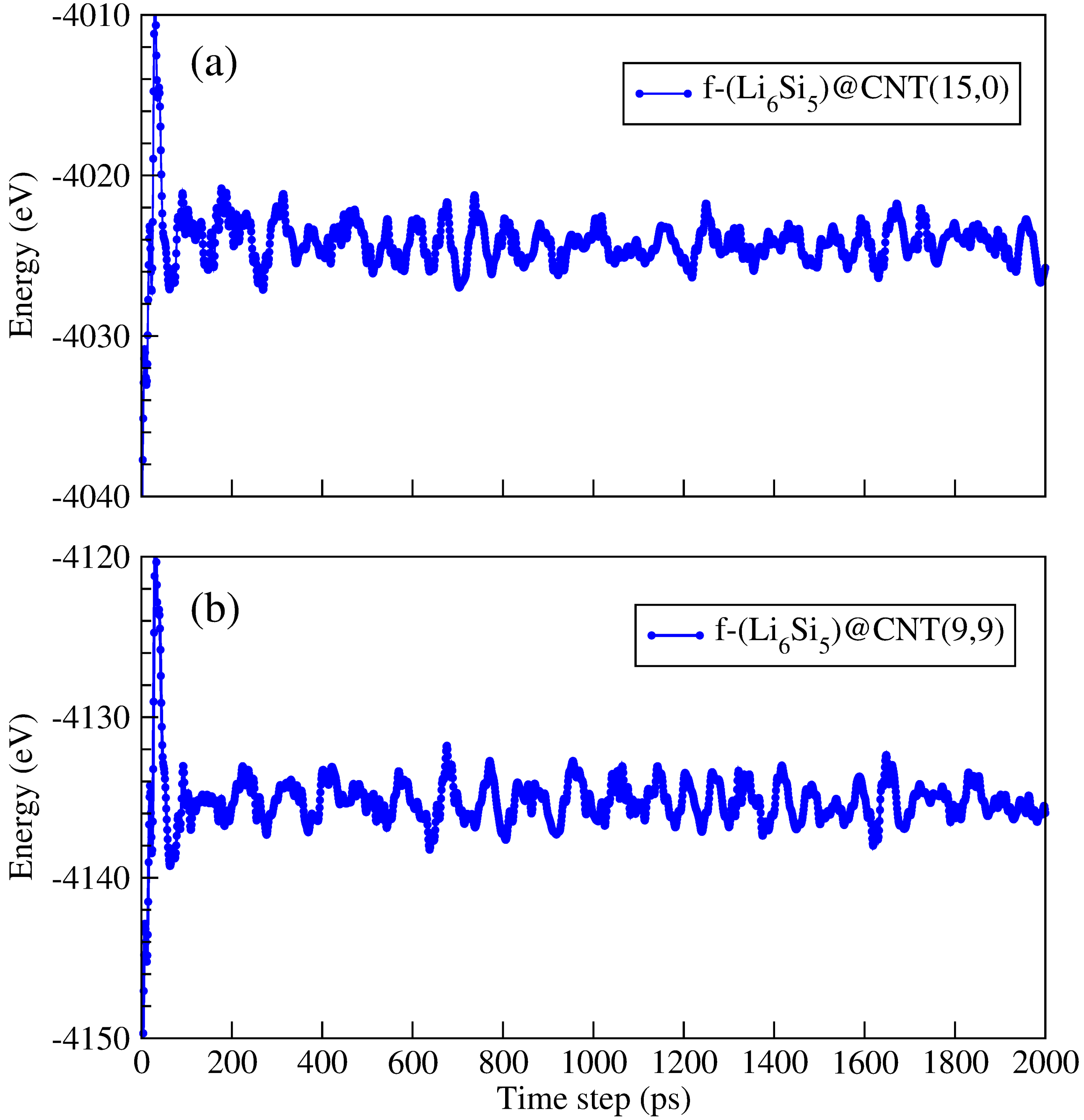


**Figure S2**. Energy as a function of time for the BO-AIMD simulations at 300 K for the f-$Li_6Si_5$ structure, (a) inside the zigzag (15,0) CNT, and (b) inside the armchair (9,9) CNT.
